# Diets, stress, and disease in the Etruscan society: Isotope analysis and infantile skeletal palaeopathology from Pontecagnano (Campania, southern Italy, 730–580 BCE)

**DOI:** 10.1371/journal.pone.0302334

**Published:** 2024-05-15

**Authors:** Giulia Riccomi, Rachele Simonit, Ségolène Maudet, Erin Scott, Mary Lucas, Valentina Giuffra, Patrick Roberts

**Affiliations:** 1 Department of Translational Research and New Technologies in Medicine and Surgery, Division of Paleopathology, University of Pisa, Pisa, Italy; 2 Department of Archaeology, Max Planck Institute of Geoanthropology, Jena, Germany; 3 History Department, Université du Mans, Le Mans, France; 4 The Arctic University Museum of Norway, Tromsø, Norway; 5 Max Planck Institute of Geoanthropology, isoTROPIC Research Group, Jena, Germany; Goethe University Frankfurt: Goethe-Universitat Frankfurt am Main, GERMANY

## Abstract

Susceptibility to morbidity and mortality is increased in early life, yet proactive measures, such as breastfeeding and weaning practices, can be taken through specific investments from parents and wider society. The extent to which such biosocialcultural investment was achieved within 1^st^ millennium BCE Etruscan society, of whom little written sources are available, is unkown. This research investigates life histories in non-adults and adults from Pontecagnano (southern Italy, 730–580 BCE) in order to track cross-sectional and longitudinal breastfeeding and weaning patterns and to characterize the diet more broadly. Stable carbon and nitrogen isotope analysis of incrementally-sampled deciduous and permanent dentine (n = 15), bulk bone collagen (n = 38), and tooth enamel bioapatite (n = 21) reveal the diet was largely based on C_3_ staple crops with marginal contributions of animal protein. Millet was found to play a role for maternal diet and trajectories of breastfeeding and feeding for some infants and children at the site. The combination of multiple isotope systems and tissues demonstrates exclusive breastfeeding was pursued until 0.6 years, followed by progressive introduction of proteanocius supplementary foods during weaning that lasted between approximately 0.7 and 2.6 years. The combination of biochemical data with macroscopic skeletal lesions of infantile metabolic diseases and physiological stress markers showed high δ^15^N_dentine_ in the months prior to death consistent with the isotopic pattern of opposing covariance.

## Introduction

Before the Roman dominance during the 3^rd^ BCE, the Italian peninsula consisted of a mosaic of independent socio-cultural groups. Of them, the Etruscans are recognized as some of the key forerunners of the Western Roman Empire [1]. The Etruscans exerted their influence over different parts of central and northeastern Italy, with expansion to the Campania region (southern Italy) during the 6^th^ century BCE with the foundation of cities like Capua and Pontecagnano [2–4]. Although Etruscan culture significantly influenced the development of pan-European trade networks and contributed to the emergence of Roman power in Italy, there remains a significant knowledge gap about their lives, health conditions and nutrition as little historical sources about them have survived. This makes it challenging to properly understand the impacts of economic, social and demographic changes in the Italian Peninsula during the 1^st^ millennium BCE on the people who lived through them.

To date, much of the archaeological research relating to the Etruscans has centred on the investigation of hillfort settlements [5], changes in agricultural practices [3,6], iron metallurgy [3], widespread exchanges of socially valuable or exotic materials, such as ivory and ostrich eggs, and changes in the use of funerary space [7–9]. aDNA analysis of Etruscan individuals provides insights into the genetic makeup and mobility of this civilization (e.g., [10–14]). As for the Etruscan diet, a large body of information is based on archaeobotanical and zooarchaeological research that has only recently started to provide insights into the food plants, animal husbandry strategies and fishing used by the Etruscans [15–19]. For example, archaeobotanical findings have allowed us to ascertain that Etruscan diet was based on diversified staple crops, particularly emmer (*Triticum dicoccum*), bread/naked wheat (*Triticum durum*, *Triticum aestivum* or *Siligo*), barley (*Hordeum vulgare*), rye (*Secale* sp.*)* and spelt (*Triticum spelt*) eaten in the form of flatbreads, soups, and porridge (puls/*pulmentum*) [3,20]. The diet also included legumes like broad beans, chickpeas, lentils, lupins beans and peas and cabbage [21,22]. The Etruscan diet also included animal terrestrial protein, in particular the consumption of sheep, goats, cattle, boar, and small wild games (birds, hare) [6,23]. Despite this, questions relating to the relationship between diet and health, and changing diet during cultural shifts and changes in social complexity in the 1^st^ milleniumc BCE, remain largely un-explored at the pre-Classical core of what would later become one of history’s largest western empires.

In this sense, cross-disciplinary work on osteoarchaeology and dietary and nutritional variability in past populations represents an important new ‘frontier’ in Classical archaeological research, offering the chance to expand the scope of archaeological investigations and to better understand how social and environmental conditions impacted health conditions [24,25]. While osteoarchaeology examines skeletal remains for signs of disease, injury, and overall life conditions, stable isotope analysis can provide clues about the nutritional status and general health of past societies called into question frailty, health and disease are not directly, nor indisputably, reflected in osteoarchaelogical assemblages. In other words, skeletal lesions in a given population may be interpreted either as a sign of frailty or as the result of a population able to live much longer with the disease to develop bony lesions (i.e., the ‘osteological paradox’ [26,27]).

In particular, the increased application of biochemical analysis to infants and children allows to reconstruct breastfeeding and weaning practices of non-survivors. This enables a better contextualization of parental investment in childcare and nutrition, sociocultural constructs, and reproductive strategy (e.g., [28,29]). The combination of palaeopathology and biochemical analysis can overcome equifinality issues [30], enabling more nuanced and plausible interpretation about food management, processing, and consumption (e.g., [31,32]) as well as to appreciate how health and diet were interconnected in past archaeological societies (e.g., [33]).

Here, we aim to incorporate previous palaeopathological observations of non-adults from the Orientalizing Etruscan period (730–580 BCE) archaeological assemblage of Chiancone II at Pontecagnano [7,34,35] that revealed evidence of metabolic diseases and physiological stress markers [36,37] with a multi-tissue isotopic approach to biochemically characterize variability in diet, feeding behaviour and early health conditions. As non-adults remains are sensitive indicators of population-level nutritional stress, δ^13^C and δ^15^N analysis of incrementally sampled permanent and deciduous teeth (n = 15) were measured to produce high-resolution data regarding breastfeeding and weaning timing and to explore physiologicalstress during tooth development. The use of the earliest-forming dentine from deciduous teeth allows us to infer maternal δ^15^N values during pregnancy [38]. We also applied δ^13^C and δ^15^N analysis of bulk bone collagen of fauna and of non-adults and adults from the same community (n = 38) and δ^13^C and δ^18^O analysis of bulk tooth enamel bioapatite of deciduous and permanent teeth of non-adults (n = 21) in order to establish standard range of dietary practices, weaning and isotopic life- history profiles in the broader osteological assemblage.

### Etruscan society during the Orientalizing period (730–580 BCE)

In comparison to other ancient cultures such as the Greeks and Romans, there is a lack of direct written sources from the Etruscan period and a series of potential biases are inherent in the existing Greek and Roman literature which reports on previous periods [39]. The primary source of knowledge about the Etruscans is therefore derived from art and archaeological findings. For example, archaeological excavations have revealed aspects of Etruscan society, including city planning, water management of agrarian territory, the discovery of temples, sacred sites, and votive offerings (e.g., [6,40–44]). Of equal importance, the exploration of famous necropolises in southern Etruria, such as Cerveteri and Tarquinia (Latium, central Italy), provides insights into funerary practices. The analysis of grave goods, including personal ornaments, weapons, vessels for food preparation and serving, as well as feasting and banqueting equipment, reveals the hierarchical nature of Etruscan society. However, this analysis provides insights into the dietary behaviors of elite members (e.g., [7,21,40,45,46]).

Our understanding regarding Etruscan society is also biased in terms of age, with adult perspective sources such as images, inscriptions, burials, and materials associated with religious practices dominating [47]. Aspects of life and health conditions among Etruscans are reconstructed from osteoarchaeological studies mostly based on adults (for an overview [48–60]). However, recent efforts have started to shed light on the social roles of non-adults, funerary practices, and the burdens of health and disease [37,61–65].

This is problematic as non-adults represent the most vulnerable members of both present and past societies as their decision making power in relation to the social and physical environment into which they are inserted is limited [66]. Infant and young child feeding practices are shaped by various cultural, religious, economic, and environmental factors, making them a complex bio-socio-cultural phenomenon (e.g., [67–69]). Therefore, investigations on children’s mortality and morbidity inform not only on dynamics of fertility but also on maternal health, cultural influences during pregnancy, wider subsistence strategies, infant rearing practices, social decisions for allocation of adequate resources and efforts made by both parents and relevant community [70–74].

### Bulk and sequential stable isotope analysis

The application of bulk stable carbon (δ^13^C) and nitrogen (δ^15^N) isotope analysis to human and animal tissues has represented a powerful tool for inferring subsistence strategies and, when combined with osteoarchaeological data, for assessing how, and to what extent, physiological and pathological processes impact human nutrition [75–82]. Stable carbon isotope (δ^13^C) variability can distinguish between plants following the two dominant photosynthetic pathways, C_3_ and C_4_, in terrestrial ecosystems [83]. In C_3_ plants, strong discrimination against the heavier isotope, ^13^C, leads to lower δ^13^C values in most of temperate vegetation, than in C_4_ plants (e.g., millet) [84]. δ^13^C values of C_3_ plants vary from c. -36.0 to -24.0‰ (global mean -26.5‰) while δ^13^C values of C_4_ plants span from c. -17.0 to -9.0‰ (global mean -12.0‰) [83]. The distinction between C_3_ and C_4_ plants is also reflected in the tissues of their consumers with small fractionation effects of 1–2‰ [85]. Stable nitrogen isotope (δ^15^N) values vary with trophic level, with shifts of +2–6‰ seen in in marine and terrestrial ecosystems [86,87], although the exact shift is variable between species and even individuals of the same species [88–90]. A greater number of trophic levels in most marine and aquatic food chains results in higher δ^15^N in marine foods and consumers compared to their terrestrial counterparts. While marine foods also have simultaneously higher δ^13^C, freshwater fish, instead, tend to have great variability in their δ^13^C due to multiple and different carbon sources compared to the terrestrial ecosystem [91–93].

The interpretation of any change in bone collagen δ^13^C and δ^15^N values as a result of food scarcity, episode of malnutrition, food shortage, metabolic or infectious disease is challenging through bulk bone collagen due to the long turnover rate and averaging of this tissue [78,94]. By contrast, the measurement of δ^13^C and δ^15^N values from incremental dentine collagen provides an avenue for exploring the intersections between diet and health, enabling correlaton between evidence of pathological conditions and isotopic stress indicators during tooth formation since teeth continue to develop even in conditions of malnutrition [38,81,85–101]. Primary dentine grows in predictable temporal increments, from the crown to the root apex, and it does not remodel after formation; therefore, signals of health conditions and dietary experiences are locked into tooth dentine [29,96,101–106].

Isotopic variation in the metabolic pathways of nitrogen and carbon can lead to anabolic and catabolic states.

The anabolic state occurs when the body needs extra protein synthesis, as in the case of growth, pregnancy, lactation, puberty, convalescence, recovery from starvation, or tissue repair (e.g., [75,78,107,108]). Research examing fast-growing tissues such as human hair recognize the anabolic profile with a decrease in δ^15^N of 0.6–2.2‰ and increase in δ^13^C values of 1.5–5.7‰ [73,80,109,110]. The catabolic state, instead, represents the general isotopic “stress pattern”, generally occurring as a result of insufficient protein due to periods of fasting, physiological stress, nutritional stress (e.g, starvation), anorexia, cachexia, hyperemesis gravidarum, cancer, infections, fevers, diarrhea, active skeletal lesions, and aging. The catabolic state is usually characterized by an increase of δ^15^N values up to 1.9‰ and parallel decrease in δ^13^C of up to 5.4‰ [76,109,111]. This means body proteins are removed faster than they are synthesized with the final result being a bone collagen δ^15^N increase in the body tissues of a stressed individual. Inadequate protein intake accompanied by energy deficiency leads to decreased carbon. Moreover, the utilization of lipids for energy, which are generally depleted in ^13^C, can further contribute to reduced δ^13^C values [25,78,88,97,98,104,109,112–115]. However, a number of studies have found little or no change in δ^13^C values during stress/disease episodes affecting δ^15^N values (e.g., [76,80,111,116–118]), suggesting that the impact of decreased protein bioavailability on carbon isotopic values is much less clear.

The application of incremental dentine isotope analysis also enables detection of breastfeeding and weaning practices in bioarchaeology. Weaning is the gradual shift from exclusive reliance on breastfeeding (from the birth mother or wet nurses) to the incorporation of complementary non-breastmilk liquids and solid foods into an infant’s diet alongside breastmilk consumption [119,120]. Generally, δ^15^N values among exclusively breastfed infants are approximately 2 to 3‰ higher than those of their mothers, since they consume their mother’s milk, a liquid tisssue with δ^15^N similar to the adult female tissues. When weaning begins with the introduction of supplementary foods, then δ^15^N decreases according to the speed and length of mixed-feeding period; once completed, δ^15^N value of the non-adults aligns with the values of the mother and other adults in the population [29,71,77,79,121–127]. Infants that are exclusively bottle fed show no enrichment [77].

While reconstruction of weaning timing through stable nitrogen isotope analysis from bulk bone collagen has some issues (due to tissue turnover rates, enrichment factors, differential isotopic composition of weaning foods compared to adult foods, on this topic (e.g., [128–130]), the application of incremental dentine is, instead, widely accepted as a routine method for more robust investigations of past breastfeeding, weaning and childhood dietary practices (e.g., [96,97,102,104,131–135]).

Finally, shifts from breastfeeding to being weaned can potentially also be tracked by applying bulk δ^13^C and δ^18^O in tooth enamel bioapatite. Oxygen isotopes are incorporated into the body through water ingestion. During breastfeeding, the δ^18^O signal is related to the amount of mother’s milk in the diet, therefore children have higher δ^18^O values which gradually decrease as they begin to consume water from isotopically ’lighter’ sources, such as the introduction of complementary foods [136]. δ^13^C values from tooth enamel bioapatite are also valuable for identifying the incorporation of supplemental foods, particularly when these foods have low protein content and are less likely to be discerned through bulk collagen carbon and nitrogen isotope signals. This approach usually relies on the comparison between the crown of first permanent molars (forming from birth to 3.5 years of age) to other permanent teeth that complete crown formation after infancy. Such comparison has a major limitation since molar amelogenesis does not incorporate an exclusive pre-weaning diet; rather, it records the late weaning period. To address this complexity, some studies (e.g. [137,138]) adopt sampling strategies that consider the crowns of deciduous teeth (forming in utero until ca. 9 months), thus reflecting exclusive/predominant breastfeeding phases, against permanent teeth to account for weaning timing. However, it should be recognized that a certain overlap (between birth-0.9 years) persists when comparing deciduous vs. permanent molars which is critical for the interpretation of weaning. One way to refine such analysis is by studying the δ^18^O values of deciduous teeth with incomplete crown formation (up to ~ 0.6 months, Cr ¾ see AlQahtani et al. [139]) in order to detetct exclusive breastfeeding.

## Materials and methods

### Archaeological site and selection of human and fauna osteological remains for stable isotope analysis

**Fig 1:**
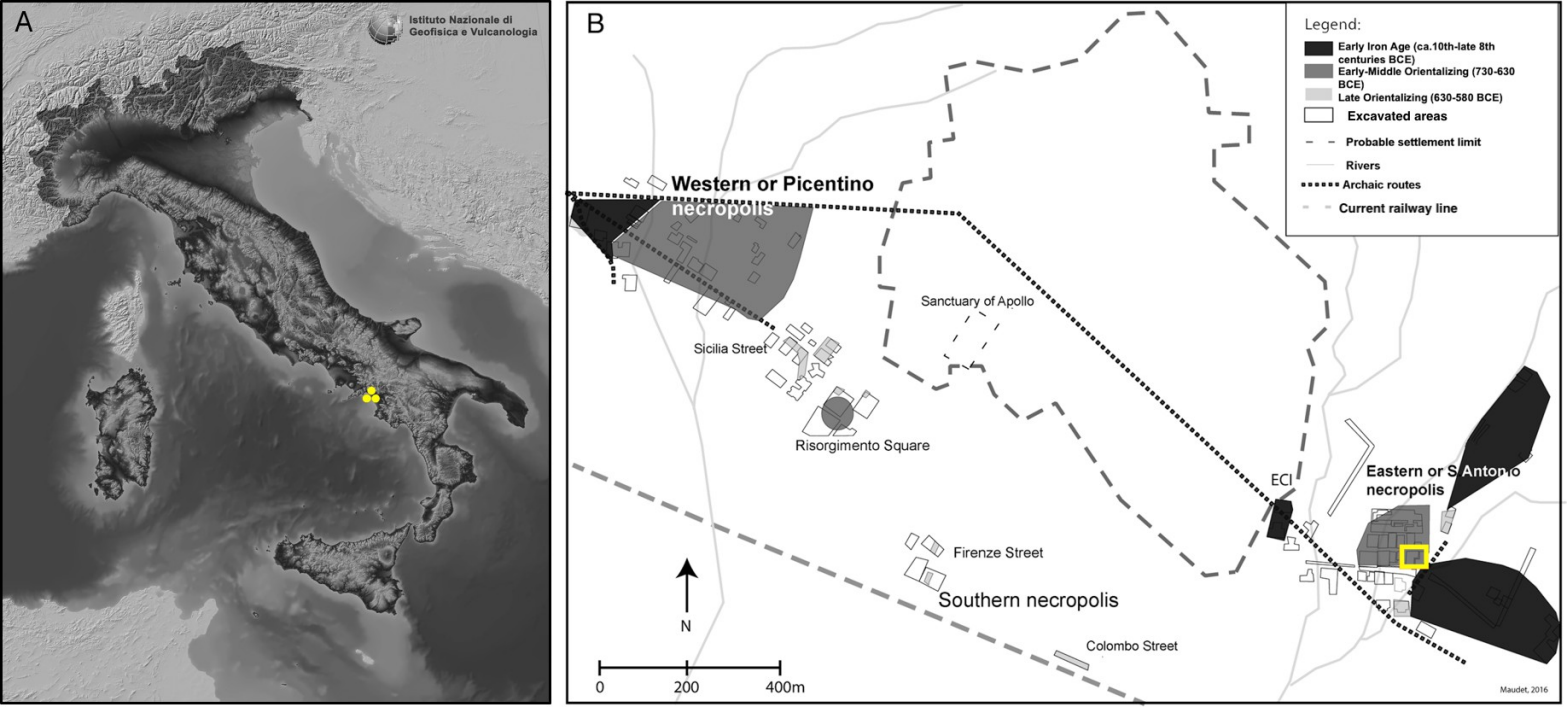
(A) The 10m-resolution TINITALY DEM with indication of the pre-Roman archaeological site of Pontecagnano (yellow dots) located in Campania region (southern Italy) (CC BY 4.0, adapted from Tarquini et al. [144]); (B) Spatial distribution of the site with indication of the settlement (dashed line), funerary areas (in grey) and location of Chiancone II funerary sector within the eastern or Sant’Antonio necropolis (yellow square) (courtesy S. Maudet).

Pontecagnano (Salerno) is one of the most important pre-Roman sites on the southern Tyrrhenian coast in Italy (Fig 1A), representing the furthest reach of the Etruscan culture in the Campania region [3,140], with the exception of Sala Consilina, a site in the Vallo di Diano who showed signs of a proto-Etruscan presence only until the end of the 8^th^ century BCE [3,4,141]. Pontecagnano was frequented by human groups from the early Iron age to the Hellenistic period (9^th^-3^rd^ centuries BCE, [44,142]). The archaeological site of Pontecagnano is situated in the Picentina plain, an alluvial plain fed by multiple mountain system rivers. This alluvial plain has various elevation shifts, forming terraced platforms (*plateaux*) as a result of surface water flow. According to Rossi [143], the presence of different types of watercourses reflects the complex environmental history of this region. Until the 20^th^ century, it was characterized by a humid environment and abundant lagoons and lake-palustrine basins that defined a marshy environment in ancient Pontecagnano. Substantial waterworks to increase arable field area and improve air quality was carried out during the Archaic age (6^th^-5^th^ centuries BCE) [44]. This particular geomorphological context has played a role in shaping not only human settlement patterns and its political and socio-economic structures, as well as health conditions, past societies in the region.

Since 1962, archaeological excavations at the site have revealed over 10,000 tombs associated with three necropolises, offering unparalleled insights into the diachronic changes of the relevant urban settlement.

The first proto-urban settlement was founded in the early Iron Age (9^th^-8^th^ century BCE) by some Villanovan groups [44,142,145], while the subsequent Etruscan Orientalizing period (730–580 BCE) witnessed intense cross-cultural interaction with Greek, Phoenician, and Italic traders, intense population growth and the emergence of powerful elite groups [3,4,146–148]. The second half of the 5^th^ century BCE saw an increasing presence of Italic groups of Samnite origin that led to a gradual cultural assimilation known as ‘Samnitisation’. The site of Pontecagnano remained a lively centre until it fell under Roman power during the 3^rd^ century BCE [44,142].

Our study pertains human skeletal remains from the funerary sector of Chiancone II located within the eastern necropolis of Pontecagnano (Fig 1B). The excavation of this part of the site took place in 1984 [7] and human skeletal remains from 73 burials of different types were uncovered. These burials consisted of simple earth-dug graves and travertine stone lined graves, with one case of burial in amphora. The analysis of grave goods (e.g., vessels and metal ornaments) allowed archaeologists to date the Chiancone II sector to the Orientalizing Etruscan period (730–580 BCE) [7,34,35]. From a sociocultural perspective, the integration of Greek artifacts and cultural practices by the Etruscans from the 8^th^ century BCE onwards drove an ideological shift toward the reorganization of social groups in funerary practices, which emphasize the presence of non-adults as a means of self-representations of the parental group [7,63,149–153]. A total of 47 individuals is currently available at the University of Pisa (Italy) represented by 29 non-adults, 9 adult males, and 9 adult females. The high representation of non-adults in the assemblage of Chiancone II makes this funerary sector particularly suitable to meet the aim of this study. Undamaged (ante- or post-mortem) permanent and deciduous teeth suitable for incremental dentine were available for 17 non-adults, while tooth crowns for tooth enamel bioapatite analysis were sampled for 21 non-adults. Additionally, teeth were selected for incremental dentine only if carious lesions were only superficially penetrating the enamel and not the underlying dentine. Bone samples from ribs (n = 31) and other skeletal elements (n = 7), with no evidence of pathological alteration, were sampled for bulk bone collagen from 38 individuals (24 non-adults and 14 adults) (Table 1). Finally, coeval faunal bones from the funerary context of Chiancone II were selected for bulk bone collagen isotope analysis (n = 5) (Table 1).

**Table 1:** List of permanent and deciduous tooth samples available for the non-adults from Pontecagnano-Chiancone II funerary sector and selected for incremental and tooth enamel bioapatite. Tooth developmental stages for permanent and deciduous teeth (*) are reported according to AlQahtani et al. [139]. Coc = cusp outline complete; Cr ½ = crown half completed with dentine formation; Cr ¾ = crown three quarters completed; Crc = crown completed with defined pulp cavity; R ¼ = root length less than crown length with visible bifurcation area; R ½ = root length equals to crown length; R ¾ = three quarters of root length developed with diverge ends; Ac = apex closed with normal periodontal ligament width. Indication of bone samples used for bulk bone collagen of non-adults and adults from the same assemblage, the latter with assigned sex and age, is also reported. Age range for non-adults refers to dental age unless specified. Skeletal pathological findings observed in the osteological assemblage are also indicated.

*ID*	*Species*	*Sex*	*Age (Range)*	Permanent Tooth* *For incremental dentine and tooth enamel bioapatite*	Deciduous Tooth* *For incremental dentine and tooth enamel bioapatite*	*Bone Sample for Bulk bone collagen*	*Skeletal/Dental Pathologies*
*PC4473*	Human	ND^a^	4.5–5.5 years	M_1_ R ¼		Rib	None
*PC4474*	Human	ND	2.5–3.5 years	M_1_ Crc		Rib	Active cribra orbitalia, cribra cranii, active SPNBF^f^; metaphyseal enlargement of long bones
*PC4475*	Human	ND	1.5–2.5 years		m_1_ R ¼	Rib	Scurvy, m_2_ superficial dental caries, alveolar resorption
*PC4476*	Human	ND	1.5–2.5 years		m_1_ R ¾ (tooth enamel bioapatite only)	Rib	Active cribra orbitalia, metaphyseal enlargement of long bones
*PC4477*	Human	ND	2.5–3.5 years		m_2_ R ½	Rib	Active cribra orbitalia, cribra cranii, cribra femoralia and humeralia; endocranial lesions; alveolar lesion; rib fracture
*PC4484*	Human	ND	6.5–7.5 years	M_1_ R ¾		Rib	LEH^g^; active SPNBF of the ilium; metaphyseal enlargement of long bones
*PC4485*	Human	ND	10+ years (non-adult)	teeth absent		Long bone	None
*PC4486*	Human	M^b^	55–65 years	teeth absent		Rib	Spina bifida occulta
*PC4488*	Human	ND	4.5 months		m_1_ Cr ½ (tooth enamel only)	Rib	Active SPNBF and metaphyseal enlargement of long bones;
*PC4490*	Human	ND	Birth		i^2^ Cr ¾ (tooth enamel only)	Rib	Active SPNBF of long bones, metaphyseal enlargement of long bones
*PC4520*	Human	ND	1.5–2.5 years		m_1_ R ½	Rib	None
*PC4527*	Human	F^c^	40–60 years			Rib	Hyperostosis frontalis interna
*PC4528*	Human	M	18–20 years			Rib	Endocranial lesions, cribra cranii
*PC4529*	Human	ND	4.5–5.5 years	M_1_ R¼		Rib	Active SPNBF of long bones and haematoma
*PC4540*	Human	M	18–24 years			Rib	None
*PC4541*	Human	ND	5.5–6.5 years	M^1^ R½		Skull	Scurvy
*PC4542*	Human	ND	neonate (40 weeks +)		teeth absent	Skull	None
*PC4545A*	Human	F	25–35 years			Rib	Vertebral abnormalities
*PC4545B*	Human	ND	neonate (2 months +)		teeth absent	Long bone	None
*PC4561*	Human	F	Adult	teeth absent		Rib	None
*PC4565*	Human	M	40–60 years			Rib	None
*PC4567*	Human	F	Adult			Rib	None
*PC4569*	Human	M	30–40 years			Rib	None
*PC4631*	Human	F	Adult			Rib	Osteomata
*PC4632*	Human	M	Adult			Rib	None
*PC4633*	Human	ND	5.5–6.5 years	M_1_ R ¼		Rib	Scurvy; LEH
*PC4634*	Human	ND	neonate (40 weeks +)		teeth absent	Long bone	None
*PC4635*	Human	ND	4.5–5.5 years	M_1_ R ¼		Rib	Active cribra orbitalia, endocranial lesions; LEH; m_1_ superficial dental caries
*PC4636*	Human	F	30–40 years			Rib	None
*PC4637*	Human	F	30–40 years			Rib	None
*PC4638*	Human	M	Adult			Rib	None
*PC4684*	Human	ND	2.5 years		m_2_ R ¼	Rib	Scurvy; fracture of the condylar process of the mandible
*PC4685A*	Human	ND	2.5 years		m_2_ R ½	\	None
*PC4685B*	Human	ND	3.5 years		m^2^ R ¾	\	Metaphyseal enlargement of long bones
*PC4687*	Human	ND	Birth-1.5 month		m_1_ Coc (tooth enamel bioapatite only)	Rib	active SPNBF and metaphyseal enlargement of long bones, endocranial lesions
*PC4688*	Human	ND	36 weeks (GA)^d^		teeth absent	Rib	Metaphyseal enlargement of long bones
*PC4689*	Human	ND	1.5–2.5 years		m^1^ R½	Rib	Scurvy
*PC4690*	Human	ND	5.5–6.5 years	M_1_ R ¼		Long bone	Cribra orbitalia with thickening of the diploë; LEH
*PC4691*	Human	ND	6.5–7.5 years	M_1_ R ½		Long bone	Cribra orbitalia; LEH, alveolar lesion
*PC4692*	Human	ND	6.5–7.5 years	M_1_ R ¾		Rib	Cribra orbitalia, LEH, active SPNBF of the long bones
***ID***	**Species**	**Sex**	**Age (range)**	**Tooth sample for bioapatite analysis**		**Bone sample for bulk bone collagen**	
*PC4474 Pig*	*Sus scrofa domesticus*	-	Non-adult	NA^e^		Rib	
*PC4477 Sheep*	*Ovis aries*	-	Adult	NA		Long bone	
*PC4520 Common pigeon*	*Columba livia domestica*	-	-	NA		Long bone	
*PC4689 Pig*	*Sus scrofa domesticus*	-	Non-adult	NA		Rib	
*PC4692 Pig*	*Sus scrofa domesticus*	-	Non-adult	NA		Rib	

Abbreviations.

^a^ ND = unknown sex.

^b^ M = male.

^c^ F = female.

^d^ GA = gestational age.

^e^ NA = sample not available.

^f^ SPNBF = subperiosteal new bone formation.

^g^ = LEH linear enamel hypoplasia.

### Osteological and palaeopathological analysis

The biological profiles of the Chiancone II non-adults and adults (i.e., age, sex, and main palaeopathological findings) were assessed in the framework of a previous Master dissertation at the University of Pisa [36] and of a focused palaeopathological research [37]. For adults, age and sex were estimated according to standard methodogical methods [154–159].

For non-adults, individuals of pre-pubertal stage, sex assessment was not performed. Whenever possible, age-at-death for non-adults included in the present research is provided (Table 1) and refers to age estimation based on dental eruption and mineralization [139] as teeth are minimally affected by environmental and nutritional stressors that may delay or accelerate the normal growth of bone tissue [160,161]. Dental age was considered most indicative of the biological age for all non-adults in this study. However, we also provide skeletal age based on long bone measurements [162] for comparison (S1 Table). Signs of skeletal lesions as listed in Table 1 were recorded for both adults and non-adults in previous research [36,37].

### Stable isotope analysis of collagen containing tissues

The skeletal remains of all individuals included in this study are held at Division of Paleopatholgy, Department of Translational Research and New Technologies in Medicine and Surgery of the University of Pisa in Italy. Identification numbers for each individual sampled are presented in Table 1. Permissions to carry out this study was issued by Direzione Regionale Musei Campania (prot. 0001763 of 2022) which complied with all relevant regulations.

Longitudinal sections of dentine from single rooted teeth and of molars were sampled according to root preparation and cleaning steps described in Beaumont et al. [104]. Each tooth was cut with a IsoMet® precision saw to obtain to equal halves. Demineralization of half of the tooth prior to 1 mm horizontal sectioning was performed using the standard modified-Longin procedure described in Richards and Hedges [163]. Teeth were partially demineralised in 0.5 M HCl at 4°C to facilitate cutting four to nine 1 mm sections from the crown to the root apex using a scalpel on half longitudinally cut demineralized teeth following method 2 from Beaumont et al. [104]. For deciduous teeth, the approximate periodic repeat interval is approximately 4 months for each increment with the first increment of crown dentine forming during fetal period [164]. For permanent teeth, each increment has an interval of approximately 9 months. In permanent dentition, the odontoblasts secrete the primary dentine of the tooth crown and root with a rate of 4–6 μm/day, while dentine mineralization at the enamel-dentine junction (EDJ) proceeds at 10–12 μm, completing crown dentinogenesis in 3–8 days. Root dentinogenesis, instead, proceeds from the cementum/dentine junction (CDJ) with a speed of 1.3–1.5 μm per day. Secondary or reparative dentine, instead, slowly accumulates throughout life with a rate of 0.4 μm/day [104,165–167].

Once the 1 mm sections were obtained, the samples were placed back in 0.5 M HCl to complete the demineralization process which took a further 18 days. Once the production of CO_2_ had ceased and the reaction was complete, all samples were rinsed three times with Milli-Q® water and placed in an HCl solution of pH3 at 70°C for 48 h to gelatinise. The solutions were filtered using Ezee filters. The resulting liquid was then freeze-dried for 48 h, weighed in duplicate and combusted in a Thermo Scientific Flash 2000 Elemental Analyser coupled to a Thermo Delta V Advantage Mass Spectrometer at the Isotope Laboratory, Max Planck Institute of Geanthropology (Jena, Germany). About 0.3 to 1 mg collagen sample was run on the mass spectrometer.

As for the extraction of bulk bone collagen, 1 g of clean bone sample was placed into 0.5 M HCl for demineralization process which last between 7 and 15 days. Once the production of CO_2_ had ceased and the reaction was complete, all samples were rinsed three times with Milli-Q® water and placed in an HCl solution of pH3 at 70°C for 48 h to gelatinise. The solutions were filtered using Ezee filters. The resulting liquid was then freeze-dried for 24 h, weighed in duplicatem and combusted in a Thermo Scientific Flash 2000 Elemental Analyser coupled to a Thermo Delta V Advantage Mass Spectrometer at the Isotope Laboratory, Max Planck Institute of Geanthropology (Jena, Germany). Preservation of archaeological collagen was evaluated following indicators of carbon and nitrogen content (%C, %N), atomic C:N ratio and collagen yield. According to Bocherens et al. [168], extraction yields in modern human bones are around 20.4 ± 3.9 wt %C. Samples containing less than 1 wt.% of collagen were considered unreliable [169]. Moreover, carbon and nitrogen contents of modern bone range from 15.3 to 47.0% and from 5.5 to 17.3%, respectively [170]. Finally, atomic C:N ratios of modern bones are generally around 3.1–3.5 [169], but can vary between 2.9 and 3.6 [171], and samples presenting values below or above these thresholds indicate alteration or contamination [169,170]. Samples that failed to meet any of these criteria were indicated in the results but excluded from statistical analysis and graphical representation.

All collagen isotopic measurements derived from dentine and bone refer to the ratio between heavy and light isotope (^13^C/^12^C or ^15^N/^14^N) measured as δ values in parts per mil (‰) calibrated using a two-point calibration between a series of International Standards (IAEA-N-2 Ammonium Sulfate: δ^15^N = +20.3 ± 0.2‰, USGS40 L-Glutamic Acid: δ^13^C = -26.389 ± 0.042‰, δ^15^N = -4.5 ± 0.1‰, IAEA-CH-6 Sucrose: δ^13^C = -10.49 ± 0.03‰, UREA Isotopic Working IRMS Standard (C^13^-N^15^): δ^13^C = -36.54; δ^15^N = -2.35 and an in-house laboratory standard (fish gelatin, δ^13^C = -15.7 and δ^15^N = 13.9). Analytical error was studied through the repeated measurement of the in-house fish gelatin standard (n = 20, ± 0.1‰ for δ^13^C and ± 0.1‰ for δ^15^N).

### Stable isotope analysis of tooth enamel bioapatite

The isotopic analysis of tooth enamel bioapatite was based on the bulk procedure described by Ventresca-Miller et al. [172]. Eight mg of tooth enamel powder from healthy permanent first and second molars from the deciduous first and second molars and lateral incisor was collected by drilling using Dremel® Micro at low speed and transferred into a 1.5 mL micro-centrifuge tubes. A 1% bleach solution (NaClO) was added for 1h to remove any organic fraction followed by rinsing with Milli-Q® water. Then, 0.1 M acetic acid was added for 10 min to remove exogenous carbonates followed by rinsing with Milli-Q® water. Micro-centrifuge tubes were sealed with Parafilm sheets, frozen and freeze-dried for four hours to remove any remaining fluid. The resulting enamel powder samples were weighed out into borosilicate glass vials (12 mL) and sealed with rubber septa.

The vials were flush filled with helium and the samples were then reacted with 100% phosphoric acid. Stable carbon and oxygen isotope analysis of the gases evolved from the samples were performed using a Thermo Gas Bench 2 connected to a Thermo Delta V Advantage Mass Spectrometer at the Max Planck Institute of Geoanthropology (Jena, Germany). The δ^13^C and δ^18^O values were calibrated (two-point calibration) using International Standards (IAEA NBS 18: δ^13^C = -5.0 ± 0.032‰, δ^18^O = -23.2 ± 0.1‰; IAEA 603: δ^13^C = +2.5 ± 0.01‰, δ^18^O = -2.4 ± 0.04‰, IAEA CO8: δ^13^C = -5.8 ± 0.032‰) and international carbonate standard (USGS44: δ^13^C = -42.1). Analytical error was studied through the repeated measurement of an in-house equid carbonate standard (n = 24, ± 0.1‰ for δ^13^C, ± 0.2‰ for δ^18^O).

### Statistics and data modelling

All data screening and processing were performed using RStudio [173]. A Shapiro Wilk test was performed in order to determine whether the fauna and human bulk bone collagen δ^13^C and δ^15^N and human tooth enamel bioapatite δ^13^C and δ^18^O were normally distributed. When data were found to be normally distributed, parametric T-Tests were used to assess whether a significant difference existed between deciduous and permanent teeth, between adults and non-adults, and between the sexes. By contrast, when the isotopic data were found to be non-normal, non-parametric Mann-Whitney U tests were used in order to assess differences. A linear regression model was used to determine if a correlation between the δ^13^C and δ^15^N values of bulk bone collagen exists. A parametric one-way ANOVA or non-parametric Kruskal-Wallis H test was used to compare mean or median values of bulk bone collagen δ^13^C and δ^15^N across different non-adult age groups and between non-adult and adult groups. In all cases, the results were considered statistically significant if the p-value was lower than 0.05.

Finally, to test whether the conventional cross-sectional approach to estimating weaning ages in archeological groups with bulk bone collagen δ^15^N values is applicable to the Pontecagnano-Chiancone II funerary sector, an approximate Bayesian computation—WARN (‘Weaning Age Reconstruction with Nitrogen isotope analysis’) R package as developed by Tsutaya and Yoneda [79] and Tsutaya [174] was applied to help estimate the beginning (t_1_) and end (t_2_) with maximum density estimators (MDE), after taking bone collagen turnover rate into consideration.

## Results

### δ^15^N and δ^13^C of faunal and human bulk bone collagen in the Orientalizing Etruscan individuals of Chiancone II

Faunal (n = 5) and human (n = 38) bone samples demonstrated satisfactory collagen quality for 4 faunal and 33 human samples based on standard %C, %N and C:N ratio criteria [168,169,171]. In total, one faunal specimen and five human samples (three non-adults and two adults) showed insufficient collagen quality, leading to their exclusion from further analysis (Tables 2 and 3).

**Table 2:** Stable carbon and nitrogen isotope values of satisfactory fauna (n = 4) and human adult (n = 12) bone collagen from the Pontecagnano-Chiancone II funerary sector.

ID	Species	Sex	Age (years)	δ^13^C (VPBD)^C^	s.d.^D^	δ^15^N (AIR)	s.d	%C	%N	C/N Ratio	%Yield
PC4486	Human	M^a^	55–65	-18.2	0.4	9.1	0.0	41.6	15.3	3.2	19.5
PC4528	Human	M	18–20	-20.5	0.1	9.0	0.0	45.9	16.0	3.3	5.2
PC4540	Human	M	18–24	-19.3	0.4	8.3	0.1	38.3	14.1	3.2	16.2
PC4569	Human	M	30–40	-20.7	0.3	9.5	0.0	38.2	13.8	3.2	7.1
PC4632	Human	M	35–45	-20.0	0.3	8.0	0.1	32.8	11.9	3.2	5.0
PC4638	Human	M	40–50	-18.8	0.1	8.6	0.1	43.2	16.2	3.1	11.6
**M_MEAN (‰) ± SD**				**-19.6 ± 1.0 ‰**		**8.8 ± 0.5 ‰**					
PC4527	Human	F^b^	40–50	-19.7	0.0	8.1	0.1	15.9	5.7	3.2	5.6
PC4545A	Human	F	25–35	-19.6	0.1	8.2	0.0	44.7	15.9	3.3	4.4
PC4561	Human	F	Adult	-19.8	0.2	7.8	0.1	40.5	14.4	3.3	4.8
PC4567	Human	F	35–55	-18.6	0.1	7.7	0.2	40.5	14.8	3.2	23.4
PC4631	Human	F	35–45	-19.7	0.1	9.4	0.0	39.8	14.5	3.2	2.2
PC4636	Human	F	30–40	-21.2	0.2	8.9	0.0	46.0	16.6	3.2	5.5
**F_MEAN (‰) ± SD**				**-19.7 ± 0.9 ‰**		**8.4 ± 0.7 ‰**					
**M+F_MEAN (‰) ± SD**				**-19.7 ± 0.9 ‰**		**8.6 ± 0.6 ‰**					
**ID**	**Species**	**Sex**	**Age**	**δ** ^**13**^ **C (VPBD)** ^**g**^	**s.d.**	**δ** ^**15**^ **N (AIR)**	**s.d.**	**%C**	**%N**	**C/N ratio**	**%Yield**
PC4474 PIG	*Sus scrofa domesticus*	-	Non-adult	-19.9	0.4	8.0	0.1	45.3	16.8	3.1	9.5
PC4520 COMMON PIGEON	*Columba livia domestica*	-	-	-21.3	0.0	5.5	0.0	44.6	16.0	3.3	9.9
PC4689 PIG	*Sus scrofa domesticus*	-	Non-adult	-20.8	0.1	5.5	0.0	33.5	11.9	3.3	8.4
PC4692 PIG	*Sus scrofa domesticus*	-	Non-adult	-19.0	0.1	6.4	0.0	27.8	9.9	3.3	10.1

Abbreviations.

^a^ M = male.

^b^ F = female.

^c^ VPDB = Vienna Pee-Dee Belemnite.

^d^ s.d. = standard deviation from duplicate results where available.

**Table 3:** Stable carbon and nitrogen isotope values of satisfactory human bone collagen of non-adults (n = 21) from the Pontecagnano-Chiancone II funerary sector.

ID	Species	Sex	Age	δ^13^C (VPBD)^C^	s.d.^D^	δ^15^N (AIR)	s.d.	%C	%N	C/N Ratio	%Yield
PC4473	Human	ND^a^	4.5–5.5 years	-19.2	0.4	8.2	0.2	37.1	13.6	3.2	4.6
PC4474	Human	ND	2.5–3.5 years	-20.1	0.2	8.5	0.0	38.1	13.6	3.3	12.4
PC4475	Human	ND	1.5–2.5 years	-19.2	0.2	8.5	0.1	31.0	11.3	3.2	11.7
PC4476	Human	ND	1.5–2.5 years	-17.9	0.1	10.2	0.0	39.8	13.9	3.3	15.9
PC4477	Human	ND	2.5–3.5 years	-19.3	0.1	8.7	0.1	32.6	11.8	3.2	29.0
PC4484	Human	ND	6.5–7.5 years	-19.1	0.1	7.6	0.1	38.9	13.4	3.4	6.9
PC4485	Human	ND	10+ years (non-adult)	-19.6	0.0	7.8	0.0	35.5	12.6	3.3	4.7
PC4488	Human	ND	4.5 months	-17.8	0.0	10.1	0.1	39.7	13.9	3.3	1.4
PC4490	Human	ND	Birth	-20.0	0.0	10.3	0.0	43.5	14.8	3.4	24.4
PC4520	Human	ND	1.5–2.5 years	-18.5	0.1	11.2	0.0	41.7	14.9	3.3	3.2
PC4529	Human	ND	4.5–5.5 years	-20.1	0.1	7.4	0.0	45.5	16.0	3.3	9.3
PC4542	Human	ND	neonate (40 weeks+)	-19.5	0.1	10.7	0.1	39.6	13.8	3.4	1.2
PC4545B	Human	ND	neonate (2 months +)	-18.5	0.1	10.2	0.1	46.0	16.1	3.3	7.0
PC4633	Human	ND	5.5–6.5 years	-20.4	0.1	9.6	0.0	45.0	16.5	3.2	11.7
PC4634	Human	ND	neonate (40 weeks +)	-20.1	0.2	8.3	0.1	40.1	14.9	3.1	8.2
PC4635	Human	ND	4.5–5.5 years	-19.3	0.0	8.0	0.0	45.1	16.4	3.2	10.1
PC4684	Human	ND	2.5 years	-20.0	0.2	8.2	0.0	42.9	15.7	3.2	11.4
PC4688	Human	ND	36 weeks (GA)^b^	-20.0	0.3	9.1	0.1	41.0	15.1	3.2	8.7
PC4689	Human	ND	1.5–2.5 years	-20.3	0.1	5.3	0.1	45.9	16.4	3.3	1.3
PC4691	Human	ND	6.5–7.5 years	-20.3	0.1	9.2	0.0	41.8	15.2	3.2	6.6
PC4692	Human	ND	6.5–7.5 years	-19.2	0.1	8.0	0.1	24.7	8.9	3.2	9.3

Abbreviations.

^a^ ND = unknown sex.

^b^ GA = gestational age.

^c^ VPDB = Vienna Pee-Dee Belemnite.

^d^ s.d. = standard deviation from duplicate results where available.

δ^13^C and δ^15^N isotopic measurements for the three omnivore samples (*Sus scrofa domesticus*) range from -20.8‰ to -19.0‰ (mean -19.9 ± 0.9‰) and from 5.5‰ to 8.0‰ (mean 6.6 ± 1.3‰), respectively. The stable carbon and nitrogen isotope values of the only avian sample (*Columba livia domestica*) were -21.3‰ and 5.5‰, respectively (Table 3). Satisfactory adult human isotopic measurements (n = 12) showed a mean of -19.7 ± 0.9‰ for δ^13^C (range -21.2 to -18.2‰) and of 8.6 ± 0.6 ‰ for δ^15^N (range 7.7 to 9.5‰) (Table 3). No statistical difference was attested between males (n = 6, δ^13^C -19.6‰ ± 1.0‰; δ^15^N 8.8‰ ± 0.5‰) and females (n = 6, δ^13^C -19.7‰ ± 0.9‰; δ^15^N 8.4‰ ± 0.7‰) for both δ^13^C (T test, df. 10, t = 0.34809, T crit = 2.2281, p = 0.735) and δ^15^N (T test, df. 10, t = 1.1314, T crit = 2.2281, p = 0.2843).

**Fig 2:**
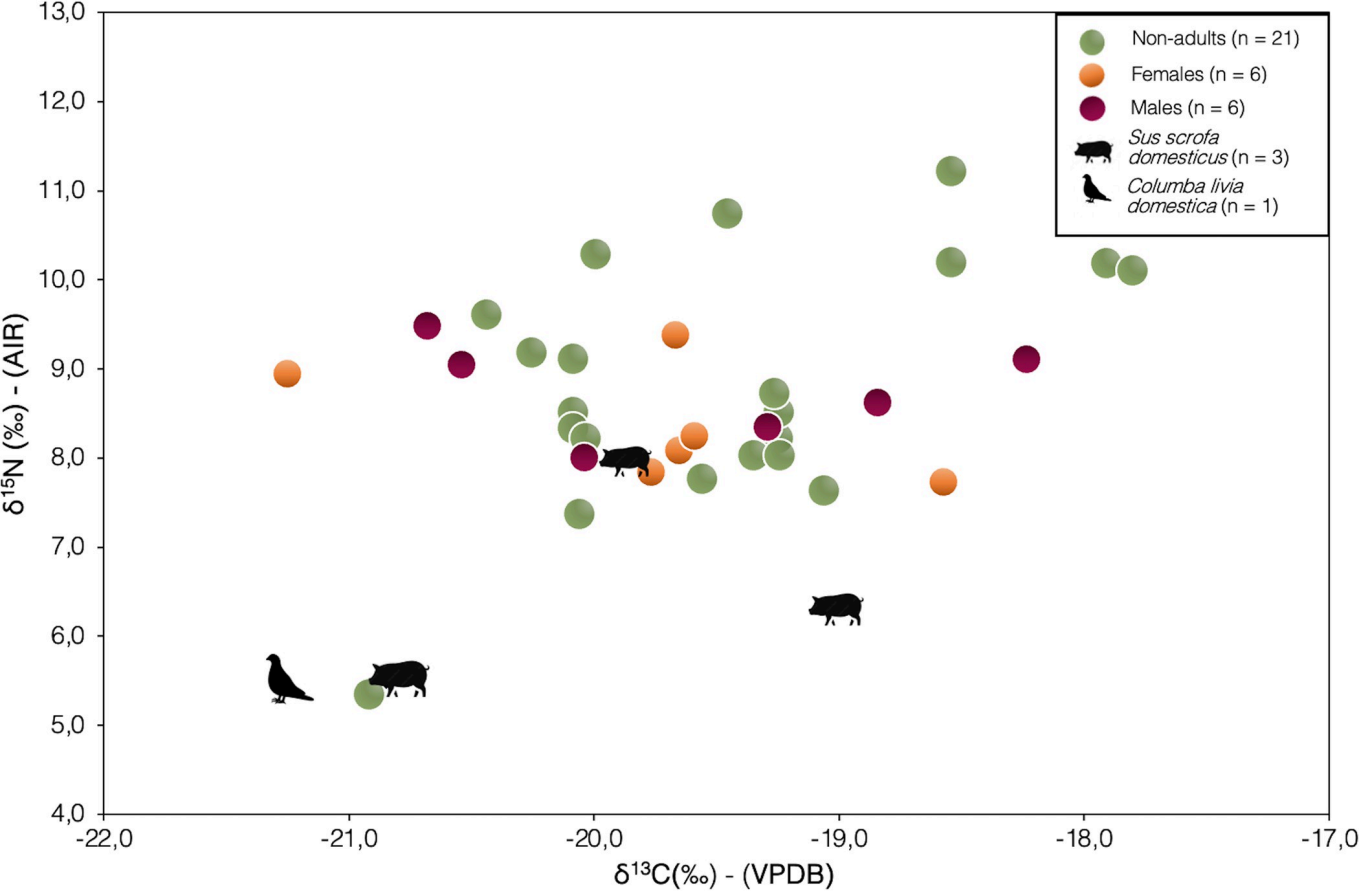
δ^13^C and δ^15^N values from human and animal bone collagen for the Pontecagnano-Chiancone II funerary sector.

Fig 2 shows the bulk adult and non-adult bone collagen values plotted alongside the fauna. In general, the human values fall just above the faunal values in terms of δ^15^N. Six non-adults with higher δ^15^N were represented by infants (PC4488, PC4490, PC4542, PC4545B) and young children (PC4476 and PC4520). One child with scurvy lesions (PC4689) has a low δ^15^N at the same level of the single avian sample and one of the three omnivores. A linear regression model of δ^13^C and δ^15^N showed very weak negative correlation between these isotopic parameters for the adult males (R = -0.188, R^2^ = 0.03549, R^2^ adjusted = -0.2056, p = 0.7208) and moderate negative correlation for the adult females (R = -0.542, R^2^ = 0.2942, R^2^ adjusted = 0.1177, p = 0.2662), respectively.

Table 4 displays the satisfactory non-adult bone collagen isotopic measurements (n = 21) arranged by age group.

**Table 4:** Stable carbon and nitrogen isotope values of human bone collagen of non-adults with satisfactory collagen parameters (n = 21) from the Pontecagnano-Chiancone II funerary sector grouped by age. Mean age used for allocation to age groups according to Cunningham et al. [162] as follows: Fetal (≤36 weeks in utero); infant (0–1 year); early childhood (2–6 years); late childhood (7–12 years). Summary statistics of stable carbon and nitrogen isotope values of non-adult and adult is also reported.

ID	Species	Sex	Age	Age Group	δ^13^C (VPBD)^C^	s.d.^D^	δ^15^N (AIR)	s.d.
PC4688	Human	ND^a^	36 weeks (GA)^b^	Fetal	-20.1	0.3	9.1	0.1
**MEAN (‰) ± SD**					**-**		**-**	
PC4488	Human	ND	4.5 months	Infant	-17.8	0.0	10.1	0.1
PC4490	Human	ND	Birth	Infant	-20.0	0.0	10.3	0.0
PC4545B	Human	ND	neonate (2 months +)	Infant	-18.5	0.1	10.2	0.1
PC4634	Human	ND	neonate (40 weeks +)	Infant	-20.1	0.2	8.3	0.1
PC4542	Human	ND	neonate (40 weeks +)	Infant	-19.5	0.1	10.7	0.1
**MEAN (‰) ± SD**					**-19.2 ± 0.9‰**		**9.9 ± 0.8‰**	
PC4475	Human	ND	1.5–2.5 years	Early childood	-19.2	0.2	8.5	0.1
PC4476	Human	ND	1.5–2.5 years	Early childhood	-17.9	0.1	10.2	0.0
PC4689	Human	ND	1.5–2.5 years	Early childood	-20.9	0.1	5.3	0.1
PC4520	Human	ND	1.5–2.5 years	Early childood	-18.5	0.1	11.2	0.0
**MEAN (‰) ± SD**					**-19.2 ± 1.3‰**		**8.8 ± 2.6‰**	
PC4474	Human	ND	2.5–3.5 years	Early childhood	-20.1	0.2	8.5	0.0
PC4477	Human	ND	2.5–3.5 years	Early childood	-19.3	0.1	8.7	0.1
PC4684	Human	ND	2.5 years	Early childood	-20.0	0.2	8.2	0.0
**MEAN (‰) ± SD**					**-19.8 ± 0.5‰**		**8.5 ± 0.3‰**	
PC4529	Human	ND	4.5–5.5 years	Early childhood	-20.1	0.1	7.4	0.0
PC4473	Human	ND	4.5–5.5 years	Early childhood	-19.2	0.4	8.2	0.2
PC4635	Human	ND	4.5–5.5 years	Early childhood	-19.3	0.0	8.0	0.0
**MEAN (‰) ± SD**					**-19.6 ± 0.4‰**		**7.9 ± 0.4‰**	
PC4633	Human	ND	5.5–6.5 years	Early childood	-20.4	0.1	9.6	0.0
**MEAN (‰) ± SD**					**-**		**-**	
PC4484	Human	ND	6.5–7.5 years	Late childhood	-19.1	0.1	7.6	0.1
PC4691	Human	ND	6.5–7.5 years	Late childhood	-20.3	0.1	9.2	0.0
PC4692	Human	ND	6.5–7.5 years	Late childhood	-19.2	0.1	8.0	0.1
**MEAN (‰) ± SD**					**-19.5 ± 0.6‰**		**8.3 ± 0.8‰**	
PC4485	Human	ND	10 + years (non-adult)	Late childhood	-19.6	0.0	7.8	0.1
**MEAN (‰) ± SD**					**-**		**-**	
**ADULT MEAN (M+F)**					**-19.7 ± 0.9 ‰**		**8.6 ± 0.6 ‰**	

Abbreviations.

^a^ ND = unknown sex.

^b^ GA = gestational age.

^c^ VPDB = Vienna Pee-Dee Belemnite.

^d^ s.d. = standard deviation from multiple samples where available.

**Fig 3:**
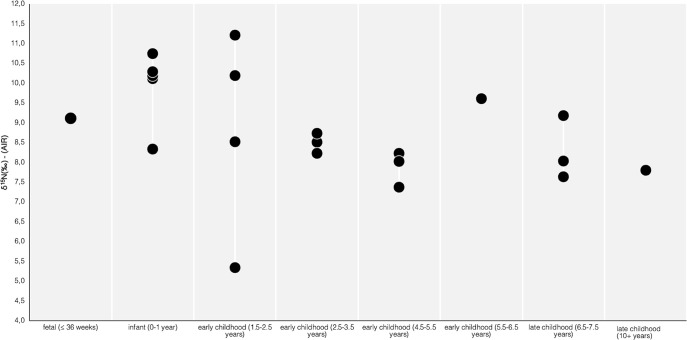
Dot plot of δ^15^N values sorted by non-adult age category.

δ^13^C means show limited changes of 1‰ across all the age groups with a range between -20.0‰ to -19.0‰. The δ^15^N mean, however, decreases from fetal and infant groups, with 9.1‰ and 9.9 ‰ respectively, to various early and late childhood groups (1.5–7.5 years) which have a δ^15^N range between 7.9‰ and 8.8‰. The unique exception is the higher δ^15^N value in the age group of 5.5–6.5 years (9.6‰) represented by a single scorbutic child (PC4633) (Table 4). Dot plot in Fig 3 shows the distribution of the δ^15^N values of non-adults (n = 21) by age group. Variability of δ^15^N values is seen between the infant (0–1 year) group (8.3–10.7‰) and late childhood (6.5–7.5 years) group (7.6–9.2‰). Marked variability can be observed in the early childhood age group (1.5–2.5 years), with a range from 5.3 to 11.2‰, which includes two individuals with scurvy lesions (PC4475 and PC4689). Differences in the mean δ^13^C and median δ^15^N values were not statistically significant across the infant, early and late childhood age groups [one-way ANOVA F(2,17) = 0.33289, p = 0.7214; Kruskal-Wallis, H test H = 5.4226, p = 0.066)], respectively.

As expected, the median nitrogen isotope values between the adult (n = 12) and infant (n = 5) groups were significantly different (Mann-Whitney test U = 6.5, z = 2.4259, p = 0.015). The same was not true for the whole early (n = 11) and late childhood groups (n = 4), however [(one-way ANOVA [F(2,24) = 0.21412, p = 0.808)] (Table 4). By contrast, the mean carbon isotope values were not significantly different when comparing the adult (n = 12) and the infant (n = 5) group [T test, df. 15, t = -1,0224, T crit 2,1314, p = 0.3228] and when comparing the adults (n = 12) with the whole early childhood age category (n = 11) and late childhood age category (n = 4) [one-way ANOVA (F(2,24) = 0.086876, p = 0.9171] (Table 4).

### Validity of weaning age estimation using the WARN model

At Pontecagnano-Chiancone II funerary sector, the bulk collagen δ^15^N values of infants and children (n = 20) were compared to those of the adult females (n = 6). The result returned from the WARN model suggested that non-adults at the site started weaning at 0.2 years of age and were completely weaned by 3.6 years (joint probability of 0.0012). However, as only estimations with joint probability >0.0025 are deemed valid, the Pontecagnano-Chiancone II estimation was considered invalid. Following adjustments to the WARN model by excluding two outliers having the highest (child PC4520) and the lowest (child PC4689) bone collagen nitrogen values (S1 File), then the model generated valid results for estimating weaning timing at the site with t_1_ at 0.7 and t_2_ at 2.6 years (joint probability 0.005).

### δ^15^N and δ^13^C of incremental dentine in the Orientalizing Etruscan non-adults of Chiancone II

Most of the incremental collagen samples analyzed showed signs of good preservation (Table 5), although C:N ratio quality parameter precluded the inclusion of some dentine increments of deciduous teeth for non-adults PC4520, PC4684, PC4685A, and PC4685B. For the same individuals, few samples containing deciduous tooth root slices resulted empty once freeze-drying procedure was completed. This means poorly preserved teeth with root collagen degraded and dissolved during HCl demineralization [175], resulting completely lost for the isotopic measurement. As a result, only individuals PC4520 and PC4685B showed a meninguful number of well-preserved increments out of the total sections of deciduous teeth (3/6 and 5/7 respectively) for graphical representation.

**Table 5:** Carbon and nitrogen isotope ratios of satisfactory incremental dentine sections from 15 non-adults buried in the Pontecagnano-Chiancone II funerary sector sorted by deciduous and permanent teeth. Note that collagen yield is calculated considering the weigh of whole tooth section.

ID	Age in years	δ^13^C (‰)	δ^15^N (‰)	%C	%N	C:N	%Yield
PC4475 M_1_−1	-0.2	-18.0	10.3	42.0	15.0	3.3	
PC4475 M_1_−2	0.2	-18.1	10.2	41.1	14.7	3.3	
PC4475 M_1_−3	0.6	-18.2	10.1	42.1	14.9	3.3	
PC4475 M_1_−4	1.0	-18.1	10.2	41.9	14.8	3.3	
PC4475 M_1_−5	1.3	-18.8	9.3	42.2	14.8	3.3	
PC4475 M_1_−6	1.7	-19.0	9.1	43.1	14.8	3.4	
PC4475 M_1_−7	2.1	-18.7	8.4	41.5	14.6	3.3	
PC4475 M_1_−8	2.5	-19.5	8.0	43.1	15.3	3.3	24.2
PC4477 M_2_−1	-0.2	-17.1	10.6	42.8	15.9	3.1	
PC4477 M2−2	0.7	-17.2	10.6	41.7	15.7	3.1	
PC4477 M2−3	1.7	-17.9	9.3	42.2	15.8	3.1	
PC4477 M2−4	2.6	-18.7	8.1	41.9	15.4	3.2	
PC4477 M2−5	3.5	-19.0	7.5	42.0	15.4	3.2	23.0
PC4520 M_1_−1	-0.2	-17.8	11.5	42.1	16.3	3.0	
PC4520 M_1_−2	0.3	-17.4	12.2	42.8	16.4	3.0	
PC4520 M_1_−3	0.9	-17.3	12.0	42.0	16.1	3.0	6.5
PC4685B M^2^–1	-0.2	-17.6	12.1	39.2	15.2	3.0	
PC4685B M^2^–2	0.4	-17.9	12.1	38.6	15.0	3.0	
PC4685B M2–3	1.0	-18.2	10.6	42.2	16.0	3.1	
PC4685B M2–4	1.7	-18.2	9.4	42.5	16.2	3.1	
PC4685B M2–5	2.3	-18.5	8.7	42.2	15.7	3.1	6.6
PC4689 M^1^–1	-0.2	-17.5	11.0	41.2	14.7	3.3	
PC4689 M^1^–2	0.3	-16.7	11.6	41.3	14.9	3.2	
PC4689 M^1^–3	0.9	-17.0	11.8	41.2	14.3	3.4	
PC4689 M^1^–4	1.4	-17.4	11.5	43.0	15.2	3.3	
PC4689 M^1^–5	2.0	-17.9	10.7	41.5	14.3	3.4	
PC4689 M^1^–6	2.5	-17.9	10.5	41.4	14.0	3.5	8.3
**MEAN (‰) ± SD DECIDUOUS TEETH**		**-18.0** ± **0.6**	**10.3** ± **1.4**				
PC4473 M_1−_1	0.3	-18.8	11.0	35.3	13.2	3.1	
PC4473 M_1−_2	1.6	-18.7	9.0	36.8	13.5	3.2	
PC4473 M_1−_3	2.9	-19.0	8.6	37.9	13.9	3.2	
PC4473 M_1−_4	4.2	-19.0	8.3	39.5	14.5	3.2	
PC4473 M_1_−5	5.5	-19.1	8.0	37.9	13.9	3.2	7.3
PC4474 M_1−_1	0.3	-18.3	11.2	44.1	15.7	3.3	
PC4474 M_1−_2	1.4	-18.6	9.7	43.6	15.4	3.3	
PC4474 M_1−_3	2.4	-18.6	9.3	43.0	15.4	3.3	
PC4474 M_1_−4	3.5	-18.8	9.3	45.1	15.9	3.3	16.2
PC4484 M_1−_1	0.3	-18.5	10.9	42.8	15.9	3.1	
PC4484 M_1−_2	1.3	-18.9	9.3	42.6	15.9	3.1	
PC4484 M_1−_3	2.4	-18.4	8.1	41.4	15.5	3.1	
PC4484 M_1−_4	3.4	-18.2	7.6	41.5	15.7	3.1	
PC4484 M_1_−5	4.4	-18.4	7.7	42.0	15.4	3.2	
PC4484 M_1_−6	5.4	-18.3	7.8	42.0	15.0	3.3	
PC4484 M_1_−7	6.5	-18.3	7.2	38.3	14.3	3.1	
PC4484 M_1_−8	7.5	-18.8	7.9	42.1	15.4	3.2	7.3
PC4529 M_1−_1	0.3	-20.0	7.4	46.8	16.5	3.3	
PC4529 M_1−_2	2.0	-20.1	7.4	45.5	16.0	3.3	
PC4529 M_1−_3	3.8	-19.4	7.3	39.3	14.3	3.2	
PC4529 M_1−_4	5.5	-19.8	7.5	44.3	15.5	3.3	10.3
PC4541 M^1^–1	0.3	-18.2	10.9	36.6	13.8	3.1	
PC4541 M^1^–2	1.2	-19.1	9.1	29.5	10.9	3.1	
PC4541 M1–3	2.1	-19.7	8.7	29.8	10.5	3.3	
PC4541 M^1^–4	3.0	-19.4	8.1	30.0	10.6	3.3	
PC4541 M^1^–5	3.8	-18.9	8.0	36.0	13.4	3.1	
PC4541 M^1^–6	4.7	-18.9	8.1	37.2	13.8	3.1	
PC4541 M^1^–7	5.6	-18.8	7.9	37.8	13.9	3.2	
PC4541 M^1^–8	6.5	-18.4	8.0	38.5	14.0	3.2	7.8
PC4633 M_1−_1	0.3	-17.7	13.0	31.5	11.7	3.1	
PC4633 M_1−_2	1.5	-18.9	11.6	35.3	13.2	3.1	
PC4633 M_1−_3	2.8	-19.7	9.4	42.3	15.6	3.2	
PC4633 M_1−_4	4.0	-19.8	9.4	41.0	15.1	3.2	
PC4633 M_1_−5	5.3	-19.9	9.3	43.0	15.2	3.3	
PC4633 M_1_−6	6.5	-19.8	9.7	42.4	15.4	3.2	12.2
PC4635 M_1−_1	0.3	-19.0	10.8	42.0	15.2	3.2	
PC4635 M_1−_2	1.2	-18.6	11.5	42.7	15.7	3.2	
PC4635 M_1−_3	2.0	-18.0	11.7	42.7	15.7	3.2	
PC4635 M_1−_4	2.9	-17.5	10.9	42.3	15.6	3.2	
PC4635 M_1_−5	3.8	-17.8	10.3	41.9	15.2	3.2	
PC4635 M_1_−6	4.6	-17.5	10.4	42.0	15.0	3.3	
PC4635 M_1_−7	5.5	-19.0	9.2	41.7	14.3	3.4	11.3
PC4690 M_1−_1	0.3	-18.2	11.0	32.1	10.5	3.5	
PC4690 M_1−_2	1.9	-17.9	9.6	36.7	12.2	3.5	
PC4690 M_1−_3	3.4	-18.9	8.6	35.5	11.6	3.6	
PC4690 M_1−_4	5.0	-18.9	8.5	40.9	13.5	3.5	
PC4690 M_1_−5	6.5	-19.0	8.5	38.8	12.7	3.6	9.1
PC4691 M_1−_1	0.3	-17.8	11.6	43.3	16.1	3.1	
PC4691 M_1−_2	1.2	-19.2	9.7	43.5	16.2	3.1	
PC4691 M_1−_3	2.1	-19.7	9.3	43.0	16.1	3.1	
PC4691 M_1−_4	3.0	-19.7	9.6	43.6	16.2	3.2	
PC4691 M_1_−5	3.9	-19.0	8.8	43.0	15.9	3.2	
PC4691 M_1_−6	4.8	-18.4	8.6	42.7	15.7	3.2	
PC4691 M_1_−7	5.7	-18.5	8.7	42.9	15.7	3.2	
PC4691 M_1_−8	6.6	-19.0	8.9	42.9	15.5	3.2	
PC4691 M_1_−9	7.5	-19.5	8.4	41.0	13.4	3.6	12.2
PC4692 M_1−_1	0.3	-18.5	9.7	43.2	15.7	3.2	
PC4692 M_1−_2	1.3	-19.1	7.0	43.2	16.0	3.2	
PC4692 M_1−_3	2.4	-19.1	7.3	42.9	15.4	3.2	
PC4692 M_1−_4	3.4	-18.7	7.2	43.5	15.7	3.2	
PC4692 M_1_−5	4.4	-18.7	7.4	43.3	15.7	3.2	
PC4692 M_1_−6	5.4	-18.0	7.7	42.8	15.4	3.2	
PC4692 M_1_−7	6.5	-18.0	7.8	42.2	15.2	3.3	
PC4692 M_1_−8	7.5	-18.5	7.9	43.1	15.3	3.3	11.8
**MEAN (‰) ± SD PERMANENT TEETH**		**-18.8** ± **0.6**	**9.0** ± **1.4**				

δ^13^C values for the Chiancone II non-adult dentine of deciduous teeth in this study range from -19.5‰ to -16.7‰ with a mean value of -18.0 (± 0.6‰) while δ^15^N values range from 7.5‰ to 12.2‰ with a mean value of 10.3 (± 1.4‰) (n = 32 total dentine samples, Table 5). δ^13^C values for the Chiancone II non-adult dentine of permanent teeth in this study range from -20.1‰ to -17.5‰ with a mean value of -18.8 (± 0.6‰) while δ^15^N values range from 7.0‰ to 13.0 ‰ with a mean value of 9.0 (± 1.4‰) (n = 64 total dentine samples, Table 5).

**Fig 4:**
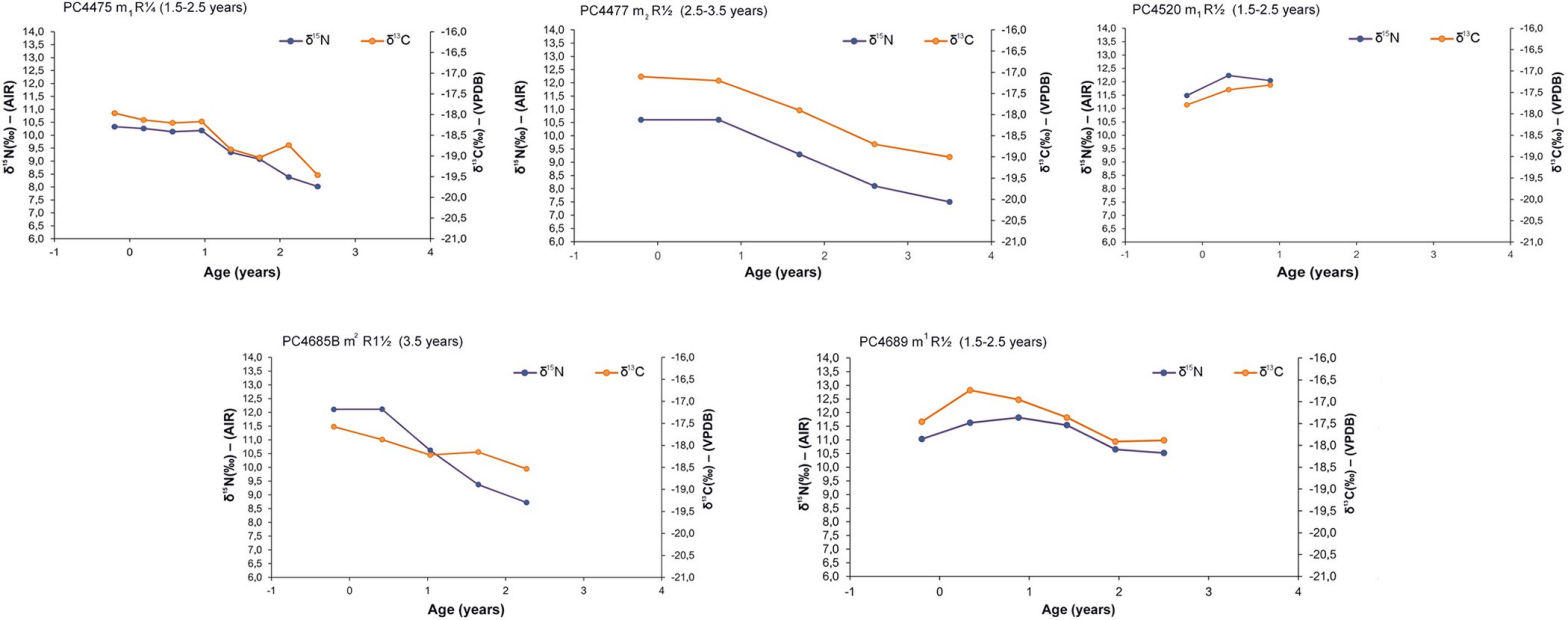
δ^13^C and δ^15^N values of dentine sections against approximate age for PC4475, PC4477, PC4520, PC4685B, and PC4689 deciduous (dec.) m^1^, m_1_ and m_2_ with relevant tooth stage development.

**Fig 5:**
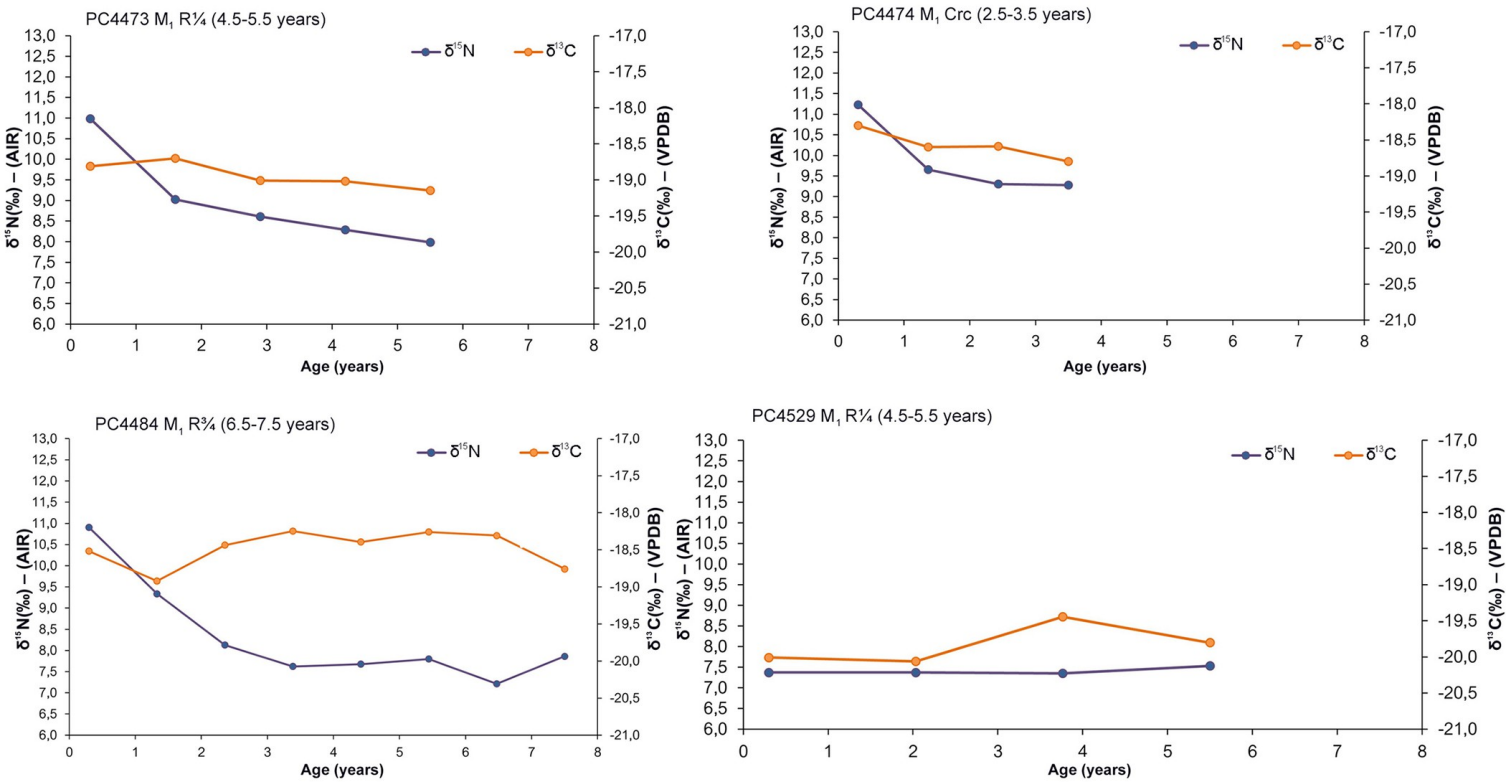
δ^13^C and δ^15^N values of dentine sections against approximate age for PC4473, PC4474, PC4484, and PC4529 permanent (perm.) M_1_ with relevant tooth stage development.

**Fig 6:**
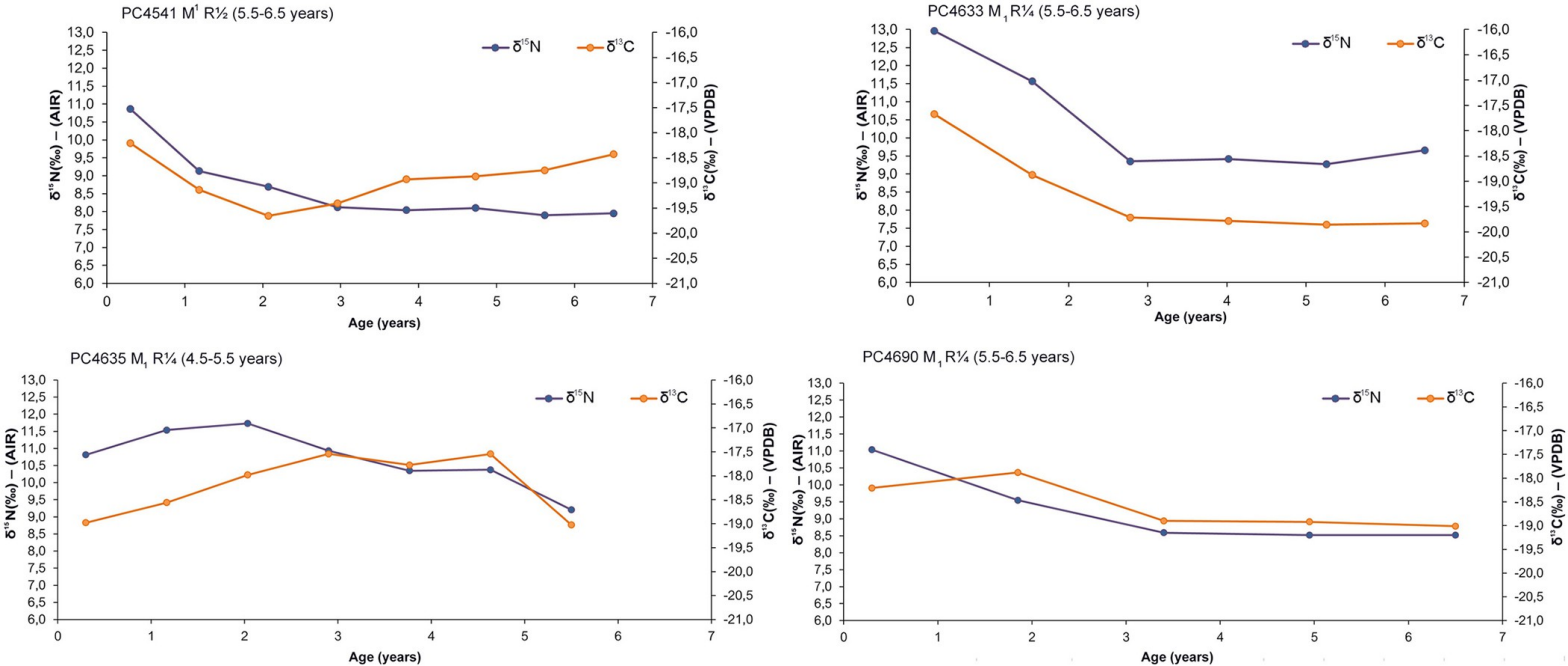
δ^13^C and δ^15^N values of dentine sections against approximate age for PC4541, PC4633, PC4635, and PC4690 permanent (perm.) M_1_ with relevant tooth stage development.

**Fig 7:**
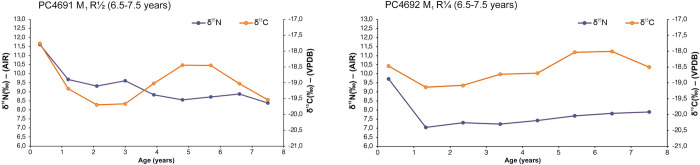
δ^13^C and δ^15^N values of dentine sections against approximate age for PC4691 and PC4692 permanent (perm.) M_1_ with relevant tooth stage development.

The results of the available incremental dentine values of deciduous teeth (n = 5) are illustrated in Fig 4 while those of permanent dentition (n = 10) are presented in Figs 5–7. Longitudinal co-varying increases and decreases in both δ^13^C and δ^15^N values, consistent with breastfeeding and weaning trophic effect were visible in 14 out of 15 incremental dentine profiles generated from deciduous and permanent teeth from the Chiancone II funerary sector, spanning approximately six months to 2.5 years (Table 6).

**Table 6:** Isotopic trends extrapolated in the incremental dentine profiles from deciduous teeth (n = 5) and permanent dentition (n = 10) from Pontecagnano-Chiancone II funerary sector.

ID	SUMMARY OF INCREMENTAL DENTINE PROFILES (DECIDUOUS TEETH)
**PC4475**	Covariance pattern of δ^13^C and δ^15^N values until ~1.7 years indicative of dietary change followed by an opposing covariance with a decrease of 0.4‰ in δ^15^N and increase of 0.8 in δ^13^C value at ~ 2.1 years of age.
**PC4477**	Concurrent decrease of δ^15^N and δ^13^C values throughout birth and infancy (-0.2–3.5 years of age) indicative of dietary change. The remarkable variability of δ^13^C values across the profile reflects both the maternal diet (first section forming in utero) and the beginning of weaning process at 0.7 years (second section) with the introduction of food enriched in δ^13^C either via maternal route or direct consumption of solid food.
**PC4520**	Co-varying increase in δ^13^C and δ^15^N values related to dietary change. First increment representing maternal diet (-0.2 years, section forming in utero) while the second and the third increment (0.3–0.9 years respectively) suggesting the introduction of food simultaneously enriched in ^13^C and δ^15^N via the maternal route, i.e., mother whilst breastfeeding, and through direct consumption of solid food.
**PC4685B**	The variability of δ^13^C values is evident, with the first increment representing maternal diet (-0.2 years, section forming in utero), and the second increment (0.4 years) possibly suggesting the introduction of food enriched in δ^13^C via the maternal route, i.e., mother whilst breastfeeding. Opposing covariance at ~ 1.7 years, followed by a concurrent decrease in δ^13^C and δ^15^N values at ~ 2.3 years and related to dietary change.
**PC4689**	Concurrent increase and decrease of δ^13^C and δ^15^N related to changes in the diet are observed across the profile. Slight variability of δ^13^C is present, with the first increment (-0.2 years, section forming in utero) representing maternal diet. The second increment (0.3 years) suggests the introduction of food enriched in δ^13^C via maternal route, i.e., mother whilst breastfeeding. The third (0.9 years) and the fourth (1.4 years) increments indicates a dietary protein source that is simultaneously enriched both in ^13^C and in ^15^N. Slight decrease of 0.2‰ in δ^15^N associated with no variation in δ^13^C values between 2.0–2.5 years of age.
**ID**	**SUMMARY OF INCREMENTAL DENTINE PROFILES (PERMANENT TEETH)**
**PC4473; PC4474; PC4690**	Co-varying decrease in δ^13^C and δ^15^N values from ~ 1.6, 1.4 and 1.9 years of age respectively until death as typical of trophic- level dietary change.
**PC4484**	Opposing covariance between ~ 2.4–6.5 years of age with the final increment at 7.5 years of age showing a rapid increase of 0.7‰ in δ^15^N and decrease of 0.5‰ in δ^13^C. LEH events at 2.6 and 3.4 years of age just as the opposing covariance pattern was began.
**PC4529**	Absence of the typical breastfeeding and weaning patterns between 0.3–3.8 years with homogeneous low flat nitrogen values with opposing covariance in the final dentine increment with increase of 0.2‰ in δ^15^N and decrease of 0.1‰ in δ^13^C.
**PC4541**	Co-varying decrease and increase in δ^13^C and δ^15^N values from 0.3 years to 5.6 years as a reflection of dietary change. Rapid increase of 0.4‰ in δ^1^3C associated with marginal decrease of 0.1‰ in δ^15^N.
**PC4633**	Concurrent decrease in δ^13^C and δ^15^N values between 0.3–2.8 years as a reflection of dietary change. Opposing covariance at ~ 5.3 years of age with rapid increase of 0.4‰ δ^15^N associated with slight variation (0.1‰) of δ^13^C the final increment.
**PC4635**	Co-varying increase and decrease in δ^13^C and δ^15^N values across the profile with marked variability of δ^13^C between 2.9- and 4.6-years suggesting incorporation of food simultaneously enriched both in ^13^C and in ^15^N.
**PC4691**	Co-varying decrease in δ^13^C and δ^15^N values between 0.3 years to 3 years of age followed opposing covariance until ~ 6.6 years; final increment exhibiting co-varying decrease in δ^13^C and δ^15^N. LEH events occurring at 2.8 and 3.4 years of age.
**PC4692**	Co-varying decrease and increase in δ^13^C and δ^15^N values until ~ 6.5 years of age as a reflection of dietary change followed by opposing covariance with rapid decrease of 0.5‰ in δ^13^C associated with very slight increase of 0.1‰ in δ^15^N value in the last increment.

In some cases, opposing covariance occurred in individuals with skeletal pathology and/or concurrently with LEH defects of permanent anterior teeth (Tables 1 and 6). However, an exact match between isotopic profile and age at onset of LEH defects is not feasible since dental defects were recorded on anterior teeth while incremental dentine profiles were generated using molars (e.g., [176]). As a summary, the isotopic trend for those who exhibited rapid δ^15^N elevation in the final dentine increment with corresponding decrease or no variation in δ^13^C were PC4484, PC4529, PC4633, and PC4692 (Figs 5–7). The isotopic trend for those who exhibited rapid δ^15^N decrease coupled with increase of δ^13^C were PC4475 and PC4541 (Figs 4 and 6). Equally important, seven individuals with several skeletal pathologies (PC4474, PC4477, PC4635, PC4685B, PC4689, PC4690, PC4691, Table 1) did not exhibit the typical ‘isotopic pattern’ of opposing covariance in the months prior their death (Figs 4 and 5).

### δ^13^C and δ^18^O of tooth enamel bioapatite in the Orientalizing Etruscan non-adults of Chiancone II

The δ^13^C and δ^18^O measurements of the non-adult teeth from Chiancone II (n = 21) are reported in Table 7.

**Table 7:** Stable carbon and oxygen isotope values of non-adult tooth enamel bioapatite of deciduous teeth (n = 11) and permanent teeth (n = 10) from the Pontecagnano-Chiancone II funerary sector. Tooth development stage is also reported according to AlQahtani et al. [139].

ID	Species	Sex	Age	Tooth	δ^13^C (‰) (VPDB)^B^	s.d.^C^	δ^18^O (‰) (VPDB)	s.d.
PC4475	Human	ND^a^	1.5–2.5 years	m_1_ R_1/4_	-8.6	0.1	-4.4	0.1
PC4476	Human	ND	1.5–2.5 years	m_1_ R _¾_	-7.9	0.2	-5.0	0.1
PC4477	Human	ND	2.5–3.5 years	m_2_ R_1/2_	-10.5	0.2	-3.6	0.1
PC4488	Human	ND	4.5 months	m_1_ Cr_½_	-7.9	0.1	-4.9	0.0
PC4490	Human	ND	Birth	i^2^ Cr¾	-11.6	0.2	-5.6	0.1
PC4520	Human	ND	1.5–2.5 years	m_1_ R_1/2_	-10.6	0.2	-2.7	0.1
PC4684	Human	ND	2.5 years	m_2_ R_1/4_	-11.3	0.2	-2.9	0.1
PC4685 A	Human	ND	2.5 years	m_2_ R_1/2_	-10.8	0.2	-3.8	0.1
PC4685 B	Human	ND	3.5 years	m^2^ R_¾_	-6.8	0.2	-4.3	0.1
PC4687	Human	ND	Birth-1.5 month	m_1_ Coc	-10.5	0.2	-6.1	0.1
PC4689	Human	ND	1.5–2.5 years	m^1^ R_1/2_	-8.6	0.2	-2.8	0.1
**MEAN (‰) ± SD DECIDUOUS TEETH**					**-9.5 ± 1.6 ‰**		**-4.2 ± 1.1‰**	
PC4473	Human	ND	4.5–5.5 years	M_1_ R_1/4_	-9.1	0.1	-4.6	0.1
PC4474	Human	ND	2.5–3.5 years	M_1_ Crc	-10.8	0.2	-4.8	0.1
PC4484	Human	ND	6.5–7.5 years	M_1_ R_¾_	-8.7	0.1	-5.3	0.1
PC4529	Human	ND	4.5–5.5 years	M_1_ R¼	-10.6	0.2	-5.1	0.2
PC4541	Human	ND	5.5–6.5 years	M^1^ R_1/2_	-10.5	0.2	-4.1	0.1
PC4633	Human	ND	5.5–6.5 years	M_1_ R _¼_	-12.3	0.2	-4.3	0.1
PC4635	Human	ND	4.5–5.5 years	M_1_ R _¼_	-7.4	0.2	-4.8	0.1
PC4690	Human	ND	5.5–6.5 years	M_1_ R_1/4_	-10.3	0.2	-3.6	0.1
PC4691	Human	ND	6.5–7.5 years	M_1_ R_1/2_	-13.9	0.2	-6.0	0.1
PC4692	Human	ND	6.5–7.5 years	M_1_ R_3/4_	-11.2	0.2	-3.9	0.2
**MEAN (‰) ± SD PERMANENT TEETH**					**-10.5 ± 1.8 ‰**		**-4.6 ± 0.7 ‰**	

Abbreviations.

^**a**^ ND = unknown sex.

^b^ VPDB = Vienna Pee-Dee Belemnite.

^c^ s.d. = standard deviation from duplicate results.

**Fig 8:**
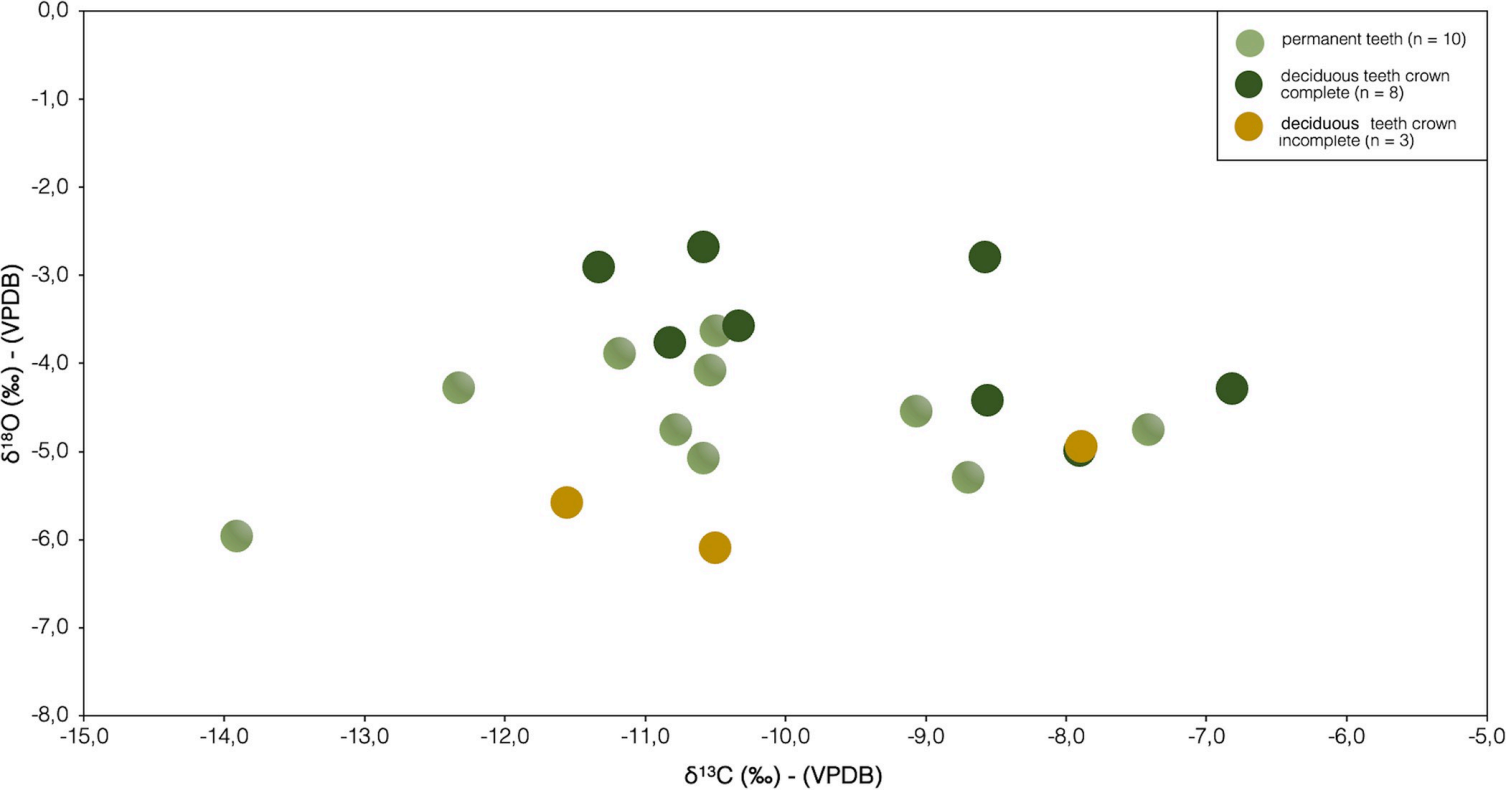
δ^13^C and δ^18^O values from human non-adult tooth enamel bioapatite for the Pontecagnano-Chiancone II funerary sector sorted by crown formation of deciduous teeth against and permanent dentition.

δ^13^C values of both permanent (n = 10) and deciduous teeth (n = 11) had a range from -13.9‰ to -7.4‰ (mean -10.5 ± 1.8‰) and from -11.6‰ to -6.1‰ (-9.5 ± 1.6‰), respectively (Fig 8). δ^18^O values have a range from -6.0‰ to -3.6 ‰ (Fig 4) (mean -4.6 ± 0.7‰, Table 7) and deciduous teeth have a range from -6.1‰ to -2.7‰ (Fig 8) (mean -4.2 ± 1.1‰, Table 7). To test whether δ^18^O values are useful for exploring breastefeeding and weaning timing in this study, data were grouped in three categories according to age-at-death of the individuals and relevant stages of crown formation (Fig 8) as follows:

- Non-adults with deciduous tooth with crown not complete (n = 3, age range birth—4.5 months) = exclusive brestfeeding;
- Non-adults with deciduous teeth with crown complete (n = 8, age range 1.5–3.5 years) = weaning process (breastmilk and supplementary foods);
- Non-adults with permanent dentition (n = 10, age range 4.5–7.5 years) = weaning complete (adult diet)

A one-way ANOVA comparing δ^13^C and δ^18^O data between the three groups showed no statistically difference for δ^13^C [(F(2,18) = 0.85309, p = 0.4426)] but did show a significant difference for δ^18^O [(F(2,18) = 7.4453, p = 0.0044)]. A post-hoc Tukey test showed that groups A and B and groups B and C differed significantly (p < 0.05).

## Discussion

### Isotopic insights from bone collagen δ^13^C and δ^15^N and tooth enamel bioapatite δ^13^C and δ^18^O into adult and non-adult Etruscan diets

The bone collagen isotope data obtained from the animals at the Ponteagnano-Chiancone II funerary sector fell within a range typical for a C_3_ environment. However, there are too few samples to determine whether the variability among the omnivorous animals (Fig 2) indicates a systematic differences in animal management, considering the estimated young age of these pigs (Tables 1 and 2, personal communication with Younes Naime). Stable isotope analysis of the bulk bone collagen of the limited number of adult indivudals at Chiancone II funerary sector revealed a diet based on the consumption of plant resources from a C_3_ ecosystem, with a limited intake of terrestrial animal protein. No preferential access to resources between adult males and females was found (Fig 2). However, the extact animal terrestrial consumption of the human group is not certain as the fauna baseline is limited in number, animal species and age of the animals whose young age could have biased the nitrogen value.

In a period of agricultural intensification, such as during the Etruscan Orientalizing period (730–580 BCE), a reliance on staple crops is not surprising. According to archaeobotanical evidence [19], a wide range of C_3_ crops (e.g., wheat, barley, oat, spelt) and pulses were consumed in the form of flatbreads, pulses, or soups (see Introduction) [20–22]. The restricted consumption of animal protein in the diet of Chiancone II individuals (Fig 2), could be interpreted in relation to socioeconomic dynamics of the Etruscan Orientalizing period. This period saw rapid population growth, long-distance trade, intensification of agriculture and the emergence of a stratified society [9,149,177]. In this context, the emergence of hierarchical groups that centralized control of the resources, based on land possession and agricultural production [3,148,178–181], might have had a direct role in food management and allocation of food sources. The archaeological interpretation of the human group inhumated at Chiancone II based on burial type, their topography and grave goods suggests that they were not representative of Orientalizing urban elites [182]; indeed, only one burial belonging to a child (PC4473) exhibited grave goods of a high level of wealth [34]. The rest of the burials had grave goods that are common for the Etruscan Orientalizing period, both in term of quantity and quality of the vessels [34]. Cuozzo [147], however, has made the hypothesis of a possible socio-economical selection in Pontecagnano necropolises, especially in the first half of the 7^th^ century BCE. With regards to, direct reflection between the society of the living and the society of the dead is not always direct, especially when considering complex urbanized communities of Iron Age and Etruscan Orientalizing period in the Campania region (e.g., [7,146,183,184]).

Our study also provides insights into non-adult Etruscan diets. The bone collagen δ^15^N value of the only non-adult skeletally classified as a fetus (PC4688) recovered from the osteological assemblage indirectly revealed the dietary input of the mother, aligning with the rest of the δ^15^N values of adults in the sample. Equally important, a fine-grained analysis of δ^15^N values of non-adults more broadly led us to consider the multifaceted biosociocultural processes represented by weaning in a given society, both in the past and today [185]. For example, four infants (PC4488, PC4490, P4542, PC4545B) and two early children aged 1.5–2.5 years (PC4476, PC4520) with the highest bone collagen δ^15^N values (Table 3) fall within the expected breastfeeding and weaning trophic level that includes milk consumption and incorporation of complementary foods as part of the weaning process (Fig 2). The completion of weaning was achieved in non-adults with an age-at-death of 2.5 years onwards, whose δ^15^N values align with those of the adult diet. This cross sectional reconstruction fits with the adjusted WARN model that indicated the beginning of weaning at 0.7 years and completion at 2.6 years of age (S1 File). Based on this approach, studies have indicated that weaning in most archaeological populations for Bronze and Iron Age Europe, across various historical, cultural, and socio-economic contexts, started around 4–6 months and was completed around 2–3 years of age, occasionally extending up to 4 years [28,79,130,138,151,186–188]. Nevertheless, despite achieving a relatively good approximation of weaning timing, the cross-sectional approach introduces challenges in interpretation. This is due to the fact that archaeological societies may not consistently depict individuals who are ’average’ or fully representative of a given group [189].

Individual PC4520 (1.5–2.5 years) exhibited the highest bone collagen δ^15^N value (Table 3) among the non-adults undergoing the weaning process, suggesting the consumption of weaned foods particularly rich in proteins, such as eggs and meat. According to secondary sources of Roman tradition [187], supplementary weaning foods included bread softened with hydromel or milk, soup made from spelt, moist porridge, and also eggs, known for their high protein content. However, as noted by Cheung et al. [130], identifying the exact nature of weaned foods in past populations is a challenging task, especially when dealing with groups for whom little or no primary written sources have survived, like the Etruscans.

In the context of linking diet to evidence for nutritional deficits or disease, the bone collagen δ^15^N values of three non-adults (PC4475, PC4684, PC4689, age range 1.5–2.5 years) with evidence of scurvy are of particular interest (Table 1, [37]). Individuals PC4475 and PC4684 exhibited δ^15^N levels consistent with the adult diet (Table 3), rather than conforming to the expected weaning trophic level. This suggests a limited intake of foods rich in proteins during the weaning process, indicating a scarcity of milk either from the mother or wet nurses, or a combination of the two with animal milk. Rib collagen of the scorbutic individual PC4689 (1.5–2.5 years) showed the lowest δ^15^N values (Fig 2), aligning with avian and one of the omnivore samples. This implies that the child’s feeding behavior relied on C_3_ resources with very limited access to animal food sources in the months prior to death. We argue that for these three scorbutic infants, sociocultural factors linked to weaning practices might have been the underlying cause for the onset of scurvy during the time of socioeconomic changes witnessed in the Etruscan Orientalizing period as discussed in a companion paper [37]. Successfully breastfed infants should not suffer from scurvy, as breast milk provides a good source of vitamin C during the first six months of postnatal life although vitamin C concentration in the milk is positively correlated to its incorporation in the maternal diet [190]. Moreover, when the introduction of complementary foods was required during weaning, the ingestion of exclusive animal milk, particularly bovine milk, is considered unhealthy as it is poor in iron and vitamin C [191], making it a potential cause of infantile scurvy. Similarly, when milk from the mother was not available, the non-adult was given boiled honey or honey mixed with goat’s milk, possibly administered through artificial nipples [187].

The combination of bone collagen with tooth enamel bioapatite from deciduous and permanent teeth provides an additional means to evaluate breastfeeding and weaning timing at the Pontecagnano-Chiancone II sector. The use of available deciduous teeth with crown formation at different stages confirms that exclusive breastfeeding occurs up to 4.5 months, as evidenced by the higher δ^18^O values in the three included infants (Fig 8), reflecting the exclusive breast milk in their diet. As supplementary foods were introduced during the weaning process, deciduous teeth with complete crowns (children aged 1.5–3.5 years) showed progressively lower δ^18^O values. Further significance is achieved when comparing this group with children aged 3.5 years and older, whose diets consist only of solid foods similar to those consumed in the adult diet. Additional insights into infant feeding practices in Etruscan society emerge when interpreting the variability of δ^13^C values in tooth enamel bioapatite. Eight non-adults of different ages had a mixed diet of C_3_ and C_4_ plants (Fig 8). The increase in δ^13^C values in tooth enamel bioapatite could be related to the introduction of a ^13^C-enriched source of carbon, such as millet and/or fish. This may occur either through the maternal route, i.e., the mother consuming millet while breastfeeding (PC4488, 4.5 months), or through the direct consumption of millet as supplementary food during weaning, gradually replacing milk for children in the age range of weaning (PC4475, PC4476, PC4685B, PC4689, 1.5–3.5 years), as well as children with complete weaning (PC4473, PC4484, PC4635, 4.5–7.5 years).

With the aim of evaluating similarities or differences with the diet at Pontecagnano-Chiancone II, we conducted comparisons with the limited available dietary isotope data from other pre-Roman groups in Italy with similar chronology. Previous stable isotope analyses of bone collagen revealed a homogeneous pattern across different Italic groups [14,192–196], whether from urban or rural contexts, indicating exclusive access to food sources within a C_3_ ecosystem with no differences by sexes or age. An exception is represented by the role of fish sources in the diet of a sample of adults from Greek colony of Metaponto (7^th^ to 2^nd^ centuries BCE, Basilicata) who showed a mixed diet with terrestrial sources from a C_3_ ecosystem integrated with marine sources [197].

### Breastfeeding and weaning in Etruscan society and childhood stress from δ^13^C and δ^15^N incremental dentine data

Regarding the analysis of the first two increments from deciduous and permanent teeth of six non-adults (PC4477, PC4520, PC4633, PC4684, PC4685B, PC4689, Figs 4–6), we can evaluate the quality of dietary input during pregnancy and/or lactation. In all these cases, δ^13^C values and their variability over the months indicate millet integration in the diet of pregnant women, subsequently transmitted to the offspring through breast milk (Table 6). According to Fuller et al. [77], δ^13^C values appear more sensitive to the introduction of solid foods, differing mainly with the photosynthetic pathways of plants in the trophic network, while δ^15^N values seem to register the duration of breast milk consumption or the trophic level effect of breastfeeding [29,77]. Other hypotheses for contextualizing the observed intra- and intertooth isotopic variability in δ^13^C values in dentine sections of non-adults at Chiancone II funerary sector involve considering variations in milk composition among mothers, between term and preterm infants, and even according to infant’s sex [198].

Furthermore, variability would be expected based on the type of breast milk produced (e.g., colostrum, transitional and mature milk). Previous research has shown changes in isotopic values of breast milk throughout the breastfeeding period, regardless of the maternal diet or health conditions [199,200]. Finally, circadian fluctuations and long-term intake of maternal lipid nutrition influence fatty acid composition of breast milk [198,201], thus contributing to variability in collagen δ^13^C values. This because carbon may be routed not only from dietary proteins but also from carbohydrates and lipids according to the macronutrient ‘scrambling model’ [29,78,202–204].

Breastfeeding, however, is time and energy consuming for the biological mother; therefore allomothering practices (both kin and non-kin) are sometimes introduced for childrearing. Figurines depicting infants with their mothers or other caregivers (Greek ‘*kourotrophos’*) suggest that the responsibility for child-rearing within the family did not rest solely with mothers [205]. Co-breastfeeding with wet-nurse and other women like neighbours, elder sisters, aunts, grand-mothers likely took place in the Etruscan world when the biological mother experienced issues with lactation (e.g., hypogalactia, new pregnancy, illnesses or death of the biological mother) [205]. In all of these cases, the isotopic values of allo-maternal breast suckling can diverge from those of the biological mother.

Direct consumption of supplementary food during weaning simoultaneously enriched in dentine δ^13^C and δ^15^N like millet and/or fish was visible in individual PC4520 at 0.9 years and PC4635 between 2.9–4.6 years (Figs 4 and 6). Dietary trajectory from the isotopic analysis of dentine increments have also helped refine the bulk bone collagen diet of the scorbutic child PC4689 (Fig 7). δ^13^C and δ^15^N values of increments between 0.9–1.4 years showed a mixed diet with the integration of millet and/or ichthyic resources along with terrestrial foods (Table 5 and Fig 4). The absence of the typical breastfeeding and weaning pattern was detected in non-adult PC4529, whose profile indicates no incorporation of breastmilk or animal milk in the diet and of weaned food rich in proteins (Fig 5).

Three non-adults (PC4473, PC4475, PC4484) with a mixed C_3_ and C_4_ diet in tooth enamel bioapatite revealed no C_4_ signal in any of their relevant dentinal increments (Table 6 and Fig 5), suggesting a more marginal role of millet as a weaned food that is visible in the ‘whole diet’ values of tooth enamel bioapatite but not captured in the more protein-biased bone collagen. Therefore, carbon isotopic values from infancy and early childhood reveal that millet had a varying role, being especially important in the diet of some pregnant and lactating women and, to a lesser extent, at the very beginning of the weaning process for a few of the non-adults. The role of millet in the human diet among pre-Roman populations in Italy is yet to be fully elucidated and this research represents an attempt to generate multitissue dietary information from human remains of Etruscan groups. These findings, represent, a major step forward in the framework of Etruscan diet, adding direct evidence of millet consumption. Archaeobotanical findings generally suffered from an artificial lack due to methodological issues in recovering small size of millet seeds rather than a real absence of this cereal in the Etruscan contexts (e.g. [19,206]).

Beyond diet, the incremental trend of non-adults from the Chiancone II sector helped us to interpret the relationship between evidence of skeletal alteration and/or disease and isotopic dietary profiles. It is well-known that not only sociocultural determinants, but also exposure to unfavorable environmental conditions, play a key role in determining the health and malnutrition status of non-adults. During the Etruscan Orientalizing period, large portions of Pontecagnano were characterized by swamps and instability of watercourses, contributing to the unhealthiness of marshy areas that favored the presence of endemic malaria and thalassemia at the site [207]. Important waterwork interventions took place only between the end of the 6th century and the beginning of the 5^th^ century BCE [44]. Wetlands are traditionally considered risky ecological settings for the spread of infectious water-borne diseases; simultaneously, proximity to water flux determines contamination, precarious hygiene standards, and inappropriate waste management [208]. All of these aspects ultimately influence human health, especially that of individuals most vulnerable like non-adults. All the non-adults included in this study died during tooth formation and they generally exhibited a burden of skeletal lesions; 13 out of 15 non-adults with generated incremental dentine profiles displayed osseous lesions (Table 1). Four individuals had an opposing covariance pattern consistent with the experience of physiological stress (catabolic state) as they showed rapid δ^15^N elevation in the final dentine increment (i.e, in the months preceding death) with a corresponding decrease or no variation in δ^13^C (Figs 4–7).

Three non-adults (PC4484, PC4529, PC4692) exhibited pathological conditions indicative of non-specific stress (i.e., LEH cribra orbitalia, active SPNBF, metaphyseal enlargement of long bones), while non-adult PC4633 was affected by infantile scurvy (Table 1). Nevertheless, the absence of vitamin C in the diet alone would not lead to starvation or elevated δ^15^N values linked to catabolism. Clinical pediatric studies, in fact, have demonstrated normal weight gain in children experiencing vitamin C deficiency [209]. However, scurvy might still have contributed to malnutrition for various reasons; painful and bleeding gums, for instance, could have presented challenges in terms of feeding and suckling [210]. At the same time, avitaminosis C impacts collagen synthesis more broadly, reflected in the onset of metaphyseal defects of long bones visible at radiological analysis and related to the active stage of the nutritional deficiency [209]. In contrast, children PC4475 and PC4541, both affected by infantile scurvy, exhibited an opposing covariance pattern, having a rapid δ^15^N decrease coupled with an increase of δ^13^C, indicative of an anabolic state in the months prior to their death. Once adequate nutrition is resumed and/or the physiological state or disease episode is overcome, neutral carbon and nitrogen balances in the body are restored [38,75,76,211,212]. We can, therefore, hypothesize the incremental dentine profiles of these three scorbutic children reflect different stages of lesions, i.e., active versus healed stage, since the progression of scurvy-lesions observed amongst these non-adults refers to both stages [37].

Previous research has examined the impact of various healing stages of skeletal lesions through bone collagen stable isotope ratios. An increase in δ^15^N was observed in active lesions, while variability in δ^15^N and δ^13^C in fractures may be associated with different healing stages of the callus [114]. Seven individuals with multiple skeletal alterations, including active SPNBF, active cranial and post-cranial porosities, endocranial lesions, LEH, alveolar lesions, metaphyseal defects (PC4474, PC4477, PC4635, PC4685B, PC4690, PC4691, Table 1), and scurvy (PC4689, Table 1), did not exhibit the expected typical ’isotopic pattern’ of opposing covariance consistent with physiological stress in the months prior to death. Experiments on various animal tissues have suggested a threshold level of nutritional stress below which isotopic changes in δ^15^N and δ^13^C are likely to be negligible [213,214]. The internal metabolic pools of carbon and nitrogen in animals with an omnivore diet appear more complex, where not only starvation but also low-quality diets result in a high carbon-to-nitrogen ratio [215].

Existing research combining incremental dentine and observable skeletal lesions in non-adults found a general pattern of opposing covariance. For instance, Goude et al. [216] identified opposing covariance with an increase in δ^15^N values and a decrease in δ^13^C between 11.5 to 14 years in an individual from Neolithic Italy, probably deceased due to tuberculosis. Similarly, King et al. [132] observed an isotopic stress pattern of opposing covariance in individuals with bone lesions associated with metabolic disorders, including scurvy, from the Atacama Desert (northern Chile) belonging to pre-agricultural (Archaic 4000–1700 BCE) and agricultural (Late Formative Period 1700 BC-450 CE) periods. In the context of scurvy, Nicholls et al. [217] documented elevated δ^15^N values in the isotopic profile of a 6 to 9-month-old infant from Iron Age Slovenia (6^th^-4^th^ century BCE) affected by avitaminosis C. Crowder et al. [218] studied skeletal remains of six non-adults (<16 years) from a Gepid cemetery in Romania (4^th^-7^th^ century CE), four of whom exhibited different bone alterations and active skeletal lesions likely related to scurvy, showing elevated δ^15^N profiles before death. Kendall et al. [219] analyzed osteological remains from two Cambridgeshire cemeteries (Edix Hill and Littleport, 5^th^-7^th^ centuries CE) where large portions of the territory were occupied by marshes (Fens) known to be endemic with malaria (*P*. *vivax*). The two locations presented different levels of stress risk, with Littleport being an island community in the Fens and Edix Hill an upland site. At Littleport, four out of five individuals displayed altered isotope ratios of δ^13^C and δ^15^N, with two showing non-specific physiologic stress markers and an opposing covariance with increasing δ^15^N and decreasing δ^13^C values. One individual exhibited nutritional stress, displaying a discrepancy between dental and skeletal development with an opposing covariance showing a decrease in δ^13^C and an increase in δ^15^N at 3.5 years. Finally, O’Donoghue et al. [25] found an increase of 0.5–1.7‰ in δ^15^N in the final dentine increments of six individuals from two urban cemeteries in London (19^th^ century CE). These individuals showed skeletal lesions of chronic diseases such as rickets, tuberculosis, as well as non-specific physiological and nutritional stress like SPNBF, LEH, endocranial lesions, and cribra orbitalia.

While we acknowledge the potential efficacy of stable isotope analysis on incremental dentine sections to elucidate infant feeding practices and improve comprehension of different trajectories of childhood stress and disease among past societies compared to bulk procedures [100], it is honest to recognize temporal resolution limits associated with the horizontal sectioning method as utilized in this study. These limitations stem from the natural growth pattern of teeth, characterized by a dome-shaped anatomical structure, which contributes to the complexity of conical dentine incremental growth, involving intricate factors such as growth rates and direction [220,221]. Alternative anatomically sensitive methods have been recently published with the aim at enhancing temporal resolution [222–226]. These methods, based on micro-punches dentine sampling, along with an age-alignment scheme predicated on average growth rates for different anatomical dentine zones, revealed that horizontal increments capture multiple dentine layers, with time average for each increment being higher compared to, leading to potential errors in estimating weaning ages. Nevertheless, Kendall et al. [227] argue that time averaging is inevitable as multiple sequential layers are sampled to obtain minimum quantity, even if advanced sampling procedures are applied.

The matemathical ‘Modeling Human Dentin Serial Sectioning (MDSS) developed by Tsutaya [221] showed that true corresponding age of the sections can differ between -2 to 5 years on average from equally assigned age, (i.e. the horizoantal sectioning), with wide range, especially accounted for sections formed at older age [221]. Therefore, the reported weaning ages and relevant patterns reconstructed in the present study should be interpreted with caution, although a general good alignment was achieved when comparing the original trajectories of Pontecagnano-Chiancone II with the MDSS (S2 File).

Further research lines may be able to refine our interpretation of Etruscan groups from the Pontecagnano-Chiancone II funerary sector. For example, calcium isotope analysis is an emerging tool in the study of breastfeeding and weaning timing, as calcium isotope values vary depending on breast milk, water, and the variable nature of solid foods introduced in the diet [228]. Compound-Specific Isotope Analysis (CSIA) of δ^13^C and δ^15^N in bone collagen can provide high-resolution data on the protein contribution of different food types (cereals, terrestrial animals, and marine sources), overcoming some of the dietary equifinality issues that may arise with traditional stable isotope analysis [229]. Equally important, δ^13^C _amino acid_ and δ^15^N _amino acid_ may better elucidate not only dietary reliance from bulk diet but also the contribution of minor proteins that may be responsible for unexplained δ^13^C and δ^15^N variations [78], as well as characterize the amino acid profile of maternal milk production [199]. Finally, lipid residue analysis absorbed in ancient cooking vessels can provide information about the preparation of weaning foods, especially in relation to millet processing, as already testified in Bronze Age Asia and Europe [230], or discovery of fatty acid specific of heating ruminant milk [231].

## Conclusions

Stable isotope analysis was conducted on human bone collagen, incremental dentine, and tooth enamel bioapatite from deciduous and permanent teeth of Etruscan non-adults and adults from the Chaincone II funerary sector at Pontecagnano (Campania, southern Italy). The sample is dated to the Orientalizing period (730–580 BCE), a time marked by significant socioeconomic and cultural upheavals following demographic increase, agricultural intensification, and the rise of a stratified society in the 1^st^ millennium pre-Roman Italy. Our data reveal that the diet primarily consisted of C_3_ staple crops, with minimal contributions of animal protein, aligning with secondary sources describing the Etruscan diet as having poor diversity. Millet was identified as playing a role in maternal diet and the feeding trajectories of some infants and children at the site.

Multi-tissue stable isotope analysis indicates that exclusive breastfeeding was practiced until ca. 0.6 years, followed by the gradual introduction of weaned foods rich in proteins (e.g., egg, fish, meat), lasting between approximately 0.7 and 2.6 years. The combination of biochemical data with early life stress (e.g., cranial porosities, periosteal new bone formation) and metabolic disease (e.g, infantile scurvy) in some individuals revealed high δ^15^N_dentine_ in the months prior to death, consistent with the isotopic pattern of opposing covariance.

This study, which combines for the first time multi-tissue biochemical analysis and palaeopathological research to investigate breastfeeding, weaning and dietary practices during the 1^st^millennium BCE in Italy, contributes to the expanding literature devoted to less-explored Italic groups of whom primary written sources are often elusive or secondary sources may introduce biases in accurately narrate the micro-histories of archaeological societies preceding Roman hegemony.

## Supporting information

S1 Table Comparion between estimated dental and skeletal age for each non-adult with available diaphyseal measurements. (DOCX)

S1 File Summary results from WARN model. (DOCX)

S2 File Comparison of Modeled age range of Dentin Serial Section (MDSS) calculated with the PlotSections function of the R package MDSS [221] against the estimated equally assigned age of the horizontal dentine sectioning method originally applied in the present study. (DOCX)
