# Supplementary material for: Diets, stress, and disease in the Etruscan society: Isotope analysis and infantile skeletal palaeopathology from Pontecagnano (Campania, southern Italy, 730–580 BCE)
Source: PLoS One. 2024 May 15;19(5):e0302334. doi: 10.1371/journal.pone.0302334 (PMC11095689; doi:10.1371/journal.pone.0302334)
Supplement: S1 Table — (DOCX) [file pone.0302334.s001.docx]

**S1 Table. Comparison between estimated dental and skeletal age for each non-adult with available diaphyseal measurements.**

Diaphyseal measurements for skeletal age [163] were available for 11 of the 29 individuals in this study. Midpoint of dental and skeletal age was used to facilitate comparison and discrepancy calculation.

Remarkable difference (≥ 1 year) between dental and skeletal age was observed in 6 of the 11 individuals suggesting that they experienced burden of disease as suggested by relevant skeletal pathological findings listed in Table 1.

| **Samples** | **Estimated dental age** | **Estimated skeletal age** | **Discrepancy between skeletal and dental age** |
| --- | --- | --- | --- |
| PC4473 | 4.5-5.5 years | \ |  |
| PC4474 | 3 years | 1.8 years | 1.2 years |
| PC4475 | 2 years | 0.8 years | 1.2 years |
| PC4476 | 2 years | 0.8 years | 1.2 years |
| PC4477 | 3 years | 2.5 years | 0.5 year |
| PC4484 | 7 years | 6.8 years | 0.2 years |
| PC4485 | \ | 10+ years (non-adult) |  |
| PC4488 | 4.5 months | 2.5 months | 2 months |
| PC4490 | Birth | neonate (40 weeks +) |  |
| PC4520 | 1.5-2.5 years | \ |  |
| PC4521 | \ | 1-3 years |  |
| PC4522 | \ | 2 years |  |
| PC4529 | 4.5-5.5 years | \ |  |
| PC4541 | 5.5-6.5 years | \ |  |
| PC4542 | \ | neonate (40 weeks +) |  |
| PC4544 | \ | infant |  |
| PC4545B | \ | neonate (2 months +) |  |
| PC4633 | 5.5-6.5 years | \ |  |
| PC4634 | \ | neonate (40 weeks +) |  |
| PC4635 | 4.5-5.5 years | \ |  |
| PC4684 | 2.5 years | 1.5 year | 1 year |
| PC4685A | 2.5 years | 1.8 year | 0.7 years |
| PC4685B | 3.5 years | 2.8 year | 0.7 years |
| PC4687 | Birth-1.5 month | \ |  |
| PC4688 | \ | 36 weeks (GA)^a^ |  |
| PC4689 | 2 years | 1 year | 1 year |
| PC4690 | 5.5-6.5 years | \ |  |
| PC4691 | 7 years | 8 years | 1 year |
| PC4692 | 6.5-7.5 years | \ |  |

**Abbreviations**

^a^ GA = gestational age

^b^ SPNBF = subperiosteal new bone formation

**References**

Cunningham, C., Scheuer, L., Black, S. (2016). *Developmental Juvenile Osteology.* Academic Press. 2nd Edition.
