# Supplementary material for: Diets, stress, and disease in the Etruscan society: Isotope analysis and infantile skeletal palaeopathology from Pontecagnano (Campania, southern Italy, 730–580 BCE)
Source: PLoS One. 2024 May 15;19(5):e0302334. doi: 10.1371/journal.pone.0302334 (PMC11095689; doi:10.1371/journal.pone.0302334)
Supplement: S1 File — (DOCX) [file pone.0302334.s002.docx]

**S2 File - Summary results from WARN model**

The four WARN assumptions applied in this study are the following: 1) t_1_ (beginning of the weaning process) occurs at 0.5 years ± 0.5; 2) t_2 (_end of the weaning process_)_ occurs at 3 years of age, ± 1 years; 3) E (enrichment between breastfeeding infant and the mother) will be 1.9‰ ± 0.9; and 4) Δ^15^_adult-wnfood_ refers to the difference between δ^15^N values of the total adult food and those of the weaning food (female adult); MDE refers to the maximum density estimator of the weaning age estimation.

The assumptions 1-2 were revised by Stantis et al. [129] whereas priors 3-4 are in their original form as published by Tsutaya and Yoneda [79]. A minimum of 0.05 marginal probability and 0.0025 joint probability were established as minimum estimators for valid results [79].

The invalid joint probability of Pontecagnano-Chiancone II WARN model might be due to either 1) scarcity of non-adult data aged between 3-6 years; or 2) data quality of some of the δ^15^N values (S2 Table).

*S2 Table. WARN model results. Probabilities not valid are in italics.*

| **WARN 1** | **MDE** | **Probability** | **Range** |
| --- | --- | --- | --- |
| *t1* | 0.2 | 0.05 | 0.0-4.2 |
| *t2* | 3.6 | *0.02* | 1.0-9.1 |
| E | 0.8 | 0.11 | 0.2-1.4 |
| *Δ*15Nadult-  wnfood (‰) | +0.11 | | |
| Joint probability | *0.0012* | | |

The latter hypothesis might find confirmation according to the generated plot (S2 Figure).

*
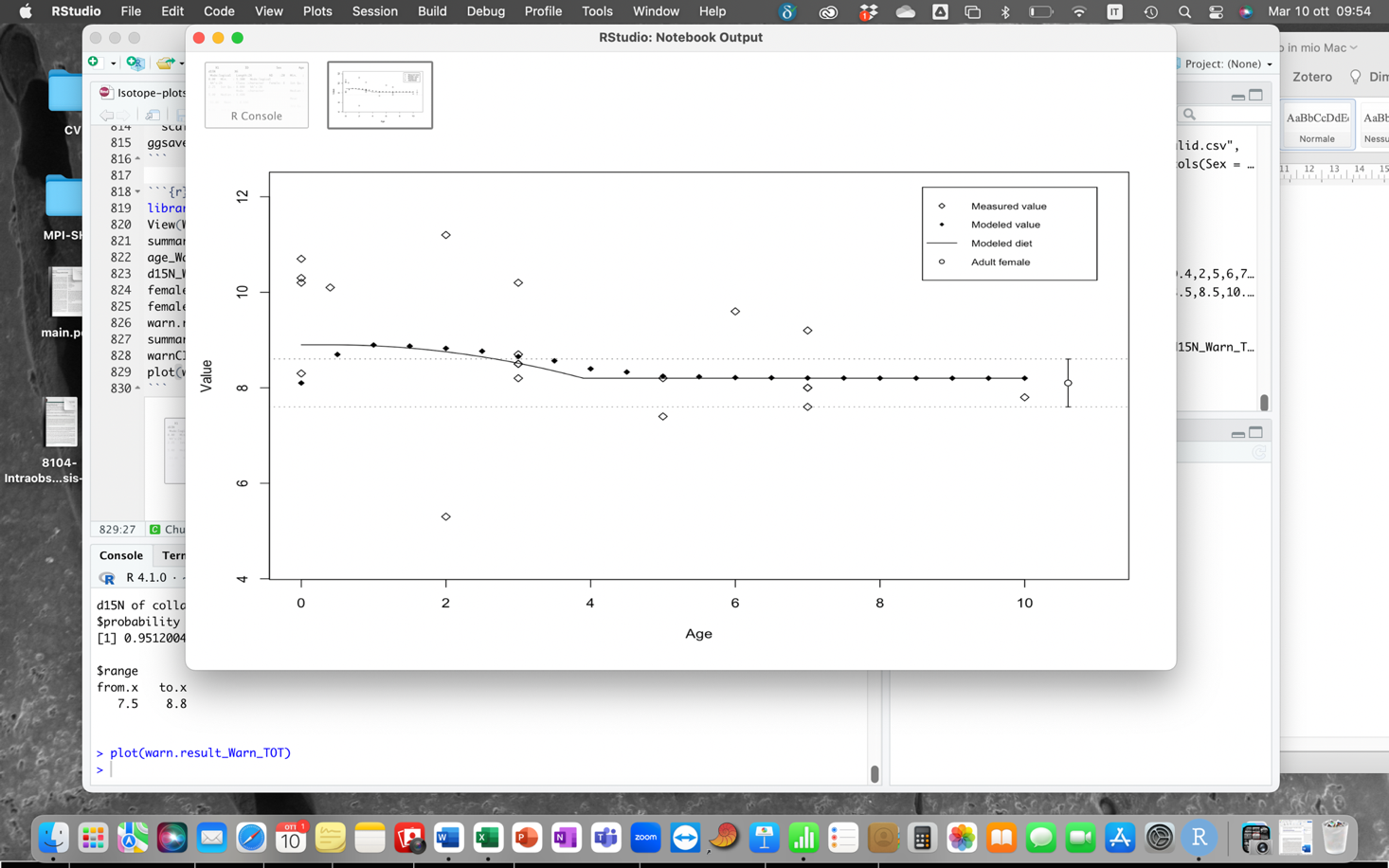
*

*S2 Figure. Graphical output generated by WARN providing invalid estimation of weaning ages at Pontecagnano-Chiancone II. Note that the model only considers non-adults as up to 10 years of age.*

The general assumption for the model is that exclusively breastfeeding infants should have the highest trophic level, and hence δ^15^N values, in a given population. Accordingly, the beginning of t_1_ can be identified where the δ^15^N values of an exclusive breastfeeding infant decline after reaching a ‘peak’, progressively declining as a reflection of the incorporation of adult foods, presumably with lower δ^15^N values. According to S2 Figure, two outliers were identified having the highest (PC4520) and the lowest δ^15^N values (PC4689), respectively. Indeed, when excluding the two outliers, then the WARN model turns to be valid (joint probability 0.005) as reported in S2.1 Table and S2.1 Figure.

*S2.1 Table. Adjusted WARN model results. Probabilities not valid are in italics.*

| **WARN 2** | **MDE** | **Probability** | **Range** |
| --- | --- | --- | --- |
| *t1* | 0.7 | 0.06 | 0.0-2.4 |
| *t2* | 2.6 | 0.08 | 0.9-4.4 |
| *E* | 2.1 | *0.04* | 0.4-3.8 |
| Δ^15^N_adult−wnfood_  (‰) | +0.16 | | |
| Joint probability | 0.005 | | |


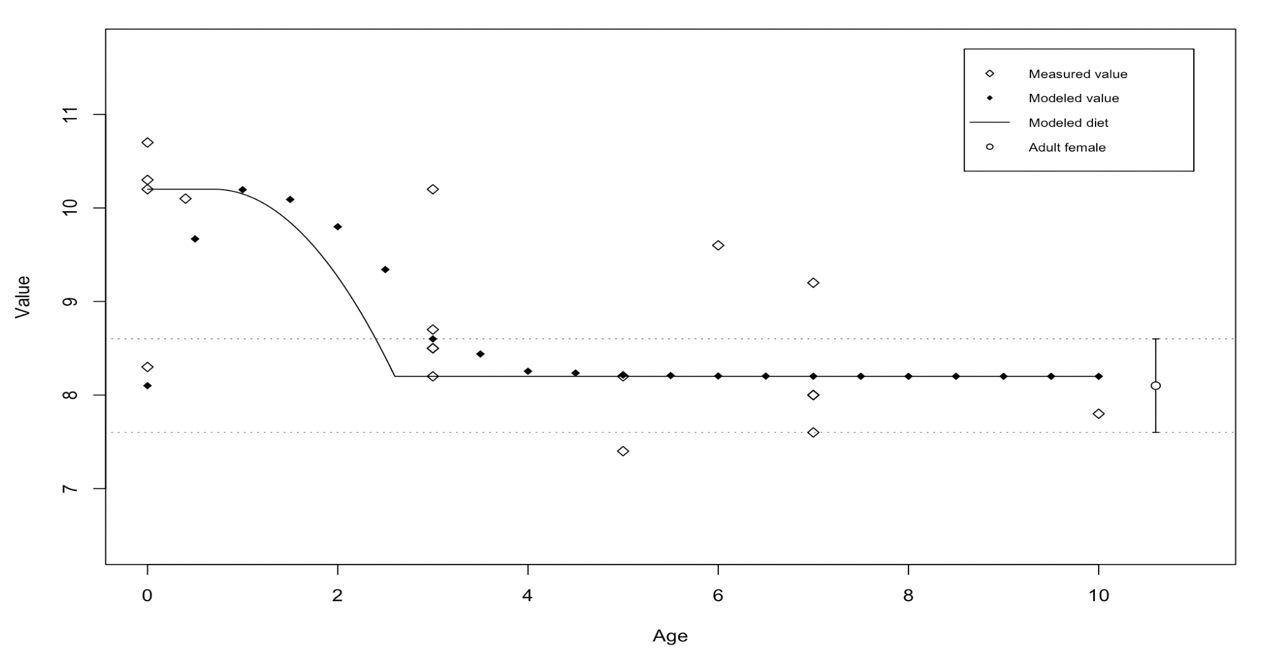


*S2.1 Figure. Exclusion of two outliers PC4520 and PC4689 produced valid estimation of weaning ages at Pontecagnano-Chiancone II. Note that the model only considers non-adults as up to 10 years of age.*
