# Supplementary material for: Diets, stress, and disease in the Etruscan society: Isotope analysis and infantile skeletal palaeopathology from Pontecagnano (Campania, southern Italy, 730–580 BCE)
Source: PLoS One. 2024 May 15;19(5):e0302334. doi: 10.1371/journal.pone.0302334 (PMC11095689; doi:10.1371/journal.pone.0302334)
Supplement: S2 File — (DOCX) [file pone.0302334.s003.docx]

**S3 File. Comparison of Modeled age range of Dentin Serial Section (MDSS) calculated with the PlotSections function of the R package MDSS [221] against the estimated equally assigned age of the horizontal dentine sectioning method originally applied in the present study**.

The original profile, estimated through equally assigned ages using the horizontal sectioning method (circle), are compared against the midpoint ages (solid black line) and age ranges (distribution) obtained from the modeled age ranges calculated using the R package MDSS (Modeled Dentin Serial Sections, [221]). There is a general good alignment between the original isotopic variability and the modeled age ranges observed across the 10 dentinal increment profiles of the permanent first molar in non-adults from the Pontecagnano-Chiancone II funerary sector.

**
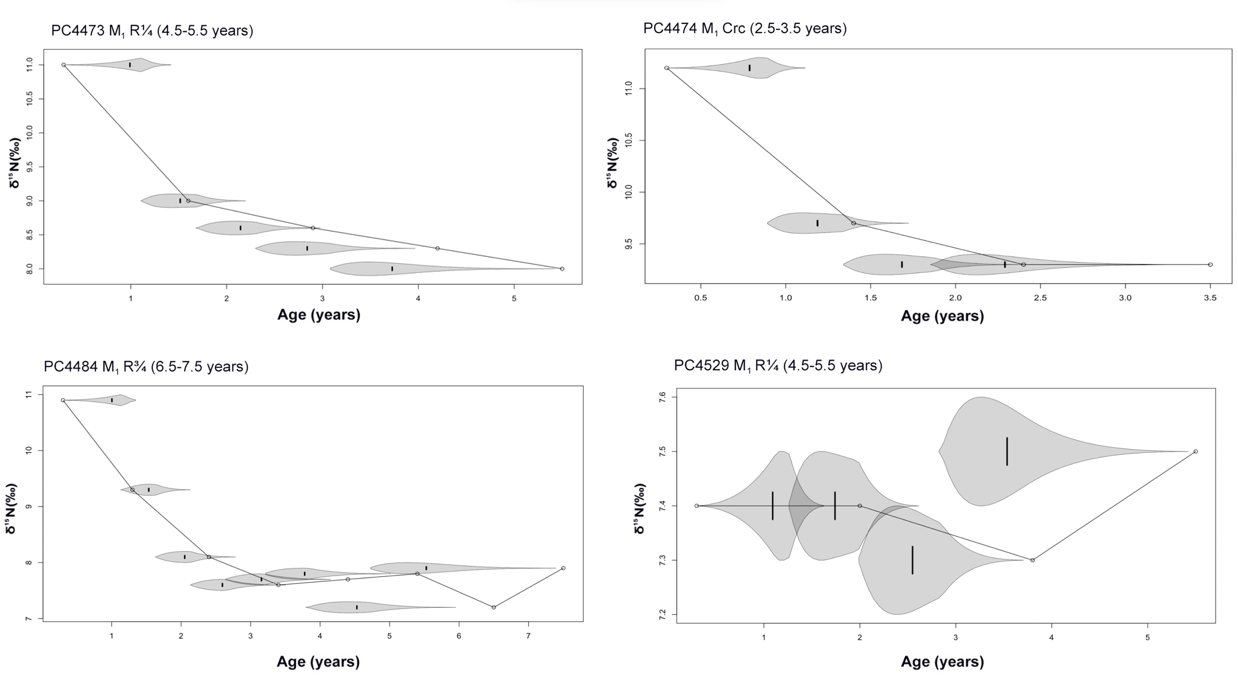
**

**
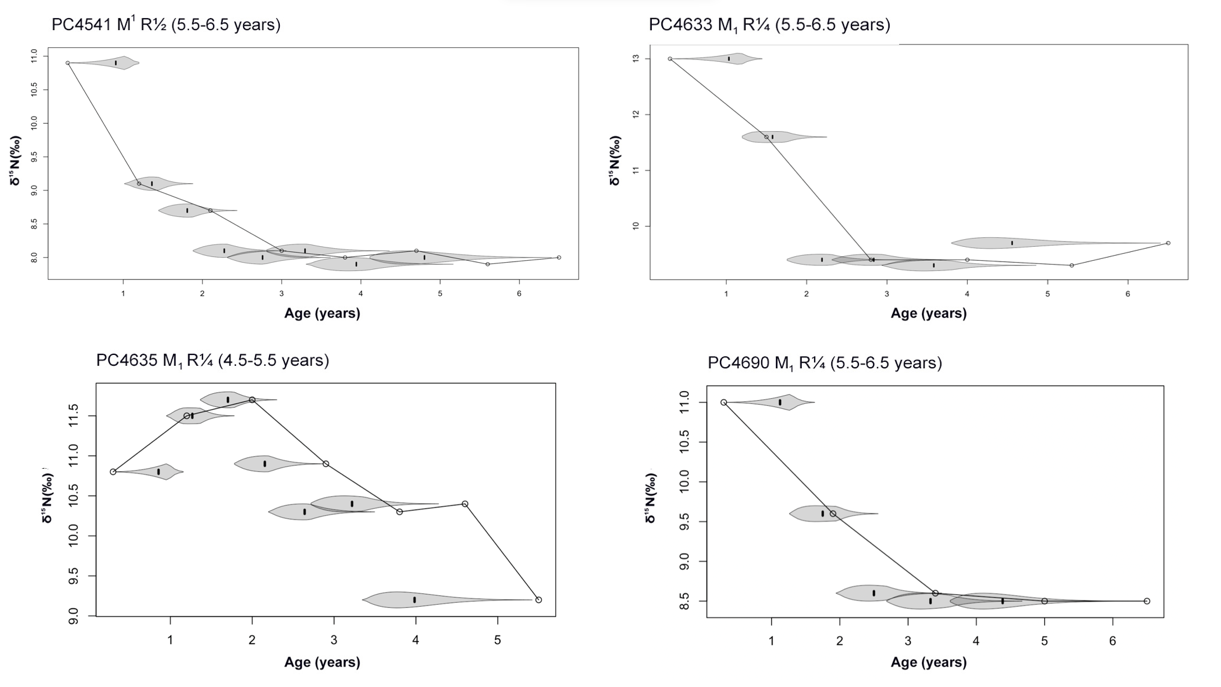
**

**
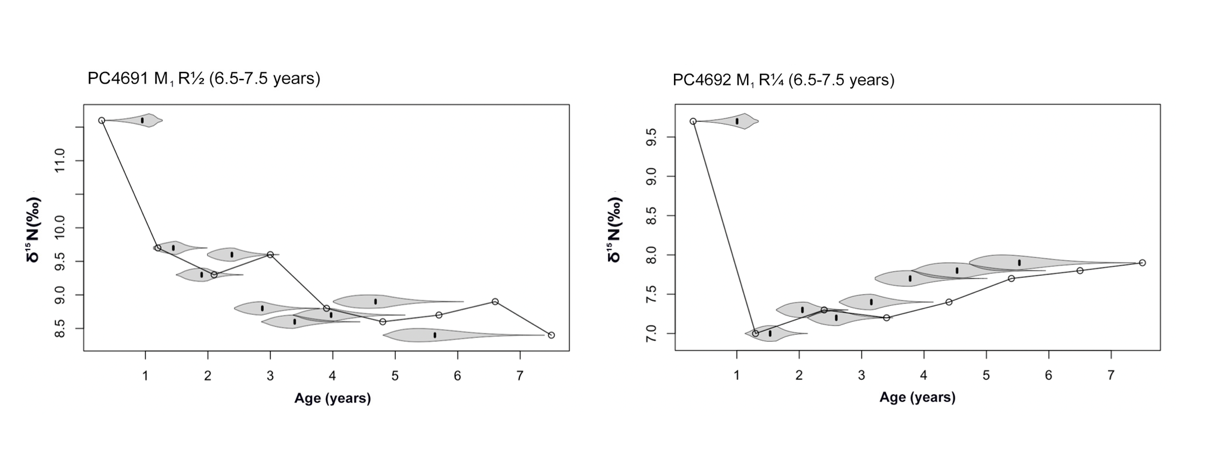
**
